# Proceedings of the 4th UK Implementation Science Research Conference: Virtual. 15-16 July 2021 https://impsciconference.com/scientific-committee/

**DOI:** 10.1186/s13012-021-01163-7

**Published:** 2021-12-20

## Institute of Psychiatry, Psychology and Neuroscience, Kings College London

### O1 Using ‘Theory-of-Change’ methodology to engage stakeholders in the co-designing of evaluation outcomes: Lessons from the implementation of mental health shared care in a Brazilian city

#### Carlos Alberto dos Santos Treichel^1^, Michelle Chanchetti Silva^1^, Rodrigo Fernando Presotto^1^, Sulamita Gonzaga Silva Amorim^1^, Maria Fernanda Lirani Dos Reis^1^, Kaleb Eliel Ferreira Leme^1^, Ugo Caramori^2^, Rosana Teresa Onocko Campos^1^

##### ^1^Department of Collective Health, State University of Campinas, Campinas, São Paulo, Brazil; ^2^Department of Internal Medicine, State University of Campinas, Campinas, São Paulo, Brazil

###### **Correspondence:** Carlos Alberto dos Santos Treichel (treichelcarlos@gmail.com)

**Background**

Stakeholder participation is understood as one of the main components of implementation research, in this sense, most studies use these subjects as informants for the process of designing integration interventions and strategies [1]. Besides, the participation of stakeholders in the process of designing the evaluation outcomes is also pointed out as a strategy that can facilitate the achievement of the expected results [2, 3]. In our study, we reported how we used the Theory-of-Change (ToC) methodology to promote stakeholder participation in this process.

**Method**

The ToC methodology included: (I) Three workshops for stakeholder input; (II) Elaboration of workshop narratives with validation by the group at the end of each workshop; (III) Elaboration of the draft ToC based on the narratives; (IV) A ToC refinement and validation workshop. The workshops were attended by 15 professionals from different backgrounds linked to specialized mental health services and Primary Care. The analysis of the narratives was guided by the analytical framework of Gadamerian hermeneutics [4].

**Results**

The preparation of the ToC was carried out after an evaluative diagnosis that made it possible to understand the functioning of the mental health network in the city. In this sense, the preparation of the ToC constituted a moment of data appropriation and consensus achievement on what would need to change for the intervention to be considered successful. As a result of this consensus, eight indicators (six quantitative and two qualitative) were developed. One indicator, divided into three dimensions, was related to the implementation outcomes; six indicators were related to service outcomes; and one indicator was related to patient outcomes.

**Conclusion**

The ToC methodology can be a powerful tool for promoting the participation of stakeholders in the design of evaluation outcomes, thus giving gr coverage and depth to the participation of these actors in the research process.

**Acknowledgments**

This work was supported by the São Paulo Research Foundation (FAPESP) through grants nº 2018/10366-6 and 2020/14309-7.

**Trial Registration**

Non applicable

**Consent to publish**

Non applicable

**References**

1. Peters DH, Adam T, Alonge O, Agyepong I, Tran N. Implementation research: what it is and how to do it. BMJ: British Medical Journal. 2013;347:f6753.

2. Furtado JP, Onocko-Campos RT. Participation, knowledge production, and evaluative research: participation by different actors in a mental health study. Cadernos de Saúde Pública. 2008;24(11):2671-2680.

3. Furtado JP, Onocko-Campos RT, Moreira MIB, Trapé TL. Participatory development of indicators for assessing mental health. Cadernos de Saúde Pública. 2013;29(1):102-110.

4. Gadamer HG. Verdade e método: traços fundamentais de uma hermenêutica filosófica. 3. ed. Petrópolis: Vozes; 2005.

### O3 Implementation findings from a hybrid type 2 pilot trial of a continuity of care model for women at risk of preterm birth in the UK

#### Cristina Fernandez Turienzo^1^, Louise H Hull^2^, Kirstie Coxon^3^, Paul T Seed^1^, Andrew H Shennan^1^, Jane Sandall^1^, on behalf of POPPIE Collaborative Group

##### ^1^Department of Women and Children’s Health, Faculty of Life Sciences and Medicine, King’s College London, London, United Kingdom; ^2^Centre for Implementation Science, Department of Health Services and Population Research, Institute of Psychiatry, Psychology & Neuroscience, King’s College London, London, United Kingdom; ^3^Department of Midwifery, Kingston University and St. George’s, University of London, United Kingdom

###### **Correspondence:** Cristina Fernandez Turienzo (cristina.fernandez_turienzo@kcl.ac.uk)

**Background**

Models of midwifery continuity of care are recommended in international guidance and at the heart of maternity policy in the UK [1-3]. However, understanding how these models work, for whom and in what context is crucial for successful implementation and scale-up [4]. POPPIE is a hybrid type 2 pilot trial conducted in South London to evaluate a care pathway that combined midwifery continuity of care and a specialist obstetric clinic for women at increased risk for preterm birth [5]. We aim to describe and evaluate the implementation, context and potential mechanisms of action, and integrate results to explore inter-relations.

**Method**

A multiphase mixed method triangulation design. Clinical outcome data were abstracted from medical records and electronic data systems. Implementation data were collected from meeting records and key documents, postnatal surveys with women (n=168) and semi-structured interviews with women, healthcare providers and stakeholders (n=53). Data from meeting records and key documents were examined narratively. Interview data were analysed using three thematic frameworks: Proctor’s [5] (for implementation outcomes), CFIR [6] (for determinants of implementation), and program theories of continuity of care [7] (for potential mechanisms). Data triangulation followed a convergent parallel and pragmatic approach which brought quantitative and qualitative data together at interpretation stage. Individual implementation measures were averaged to give a composite implementation strength score using a similar approach previously described in other studies [8,9].

**Results**

Overall, the POPPIE model was feasible, delivered with high fidelity and satisfied most women. Despite delays in early adoption delays (likely associated with lack of existing continuity models at the hospital), most midwives and clinical managers reported the model was embedded within established services and sustained and adapted after the trial (strongly facilitated by national maternal policy on continuity pathways). Potential mechanisms of impact identified included e.g. access to care, advocacy and perceptions of safety and trust. There was no correlation between implementation outcomes, or between the composite score and the primary outcome.

**Conclusion**

We demonstrated the POPPIE model was feasible, and a larger trial is needed. Measuring implementation alongside the clinical outcomes was beneficial in understanding context, potential mechanisms and results. These findings contribute to a recognised gap in the literature in the field of maternal health, by providing an example of how to integrate implementation science principles and evaluate contextual factors affecting implementation outcomes alongside clinical outcomes.

**Trial registration:**

UKCRN Portfolio Database (prospectively registered, 24 April 2017): 31951; ISRCTN registry (retrospectively registered, 21 August 2017): ISRCTN37733900.

**Consent to publish**

NA

**References**

1. NHS long term plan. Maternity and neonatal services. 2019. https://www.longtermplan.nhs.uk/online-version/chapter-3-further-progress-on-care-quality-and-outcomes/a-strong-start-in-life-for-children-and-young-people/maternity-and-neonatal-services/ Accessed 7 June 2019

2. World Health Organisation. WHO recommendations on antenatal care for a positive pregnancy experience. WHO: Geneva; 2016.

3. World Health Organisation. WHO recommendations: intrapartum care for a positive childbirth experience. WHO: Geneva; 2018

4. Sandall J, Soltani H, Gates S, Shennan A, Devane D. Midwife-led continuity models versus other models of care for childbearing women. *Cochrane Database Syst Rev*. 2016;8: CD004667

5. Proctor E, Silmere H, Raghavan R, Hovmand P, Aarons G, Bunger A et al. Outcomes for implementation research: conceptual distinctions, measurement challenges, and research agenda. *Adm Policy Ment Health.* 2011; 2:65-76.

6. Consolidated Framework for Implementation Research. CFIR Constructs. https://cfirguide.org Accessed 17 January 2019.

7. Fernandez Turienzo C, Rayment‐Jones H, Roe Y, Silverio SA, Coxon K, Shennan AH, Sandall J. A realist review to explore how midwifery continuity of care may influence preterm birth in pregnant women. *Birth*. 2021;

8. Vousden N, Lawley E, Seed PT et al. Exploring the effect of implementation and context on a stepped-wedge randomised controlled trial of a vital sign triage device in routine maternity care in low-resource settings. *Implement Sci*. 2019; 14:38.

9. Farris RP, Will JC, Khavjou O, Finkelstein EA. Beyond effectiveness: evaluating the public health impact of the WISEWOMAN program. *Am J Public Health*. 2007; 4:641–7.

### O5 Integrating a process theory and a determinant framework to understand how contextual factors, cognitive work and social processes interact to drive implementation: Methodological insights

#### Dawn Schroeder^1*†^, Thea Luig^1,2†^, Sanjay Beesoon^3^, Denise Campbell-Scherer^2,4,5^

##### ^1^Physician Learning Program, Faculty of Medicine and Dentistry, University of Alberta, Edmonton, Alberta, T6G 1C9, Canada; ^2^Family Medicine, Faculty of Medicine and Dentistry, University of Alberta, Edmonton, Alberta, T6G 1C9, Canada; ^3^Surgery Strategic Clinical Network, Alberta Health Services, Edmonton, Alberta, T5J 3E4, Canada; ^4^Office of Lifelong Learning and Physician Learning Program, Faculty of Medicine and Dentistry, University of Alberta, Edmonton, Alberta, T6G 1C9, Canada; ^5^Alberta Diabetes Institute, University of Alberta, Edmonton, Alberta, T6G 1C9, Canada

###### **Correspondence:** Dawn Schroeder (dawn.schroeder@ualberta.ca)

*Authors Dawn Schroeder and Thea Luig contributed equally to this paper and share lead authorship.

**Background**

Implementation science has largely treated the study of contextual determinants and social and cognitive processes separately. Yet, in complex healthcare spaces, it is key to understand how contextual factors interact with individual and collective work to drive implementation of an innovation. We explore the how-to and practical relevance of integrating a process theory with a determinant framework and discuss methodological insights using a case example.

**Method**

We chose Normalization Process Theory (NPT) and the Consolidated Framework for Implementation Research (CFIR) to conduct a qualitative study of the roll out of the National Surgical Quality Improvement Program (NSQIP) across several hospital sites in Alberta, Canada. We integrated NPT with CFIR to guide the development of the interview guide, coding manual, and to analyse the results.

**Results**

Integrating NPT with CFIR deepened our understandings of how cognitive work, social processes and contextual factors interact to enable or impede implementation of NSQIP. [1,2] In particular, NPT provided more granularity within the CFIR process domain to understand what work the implementation teams engaged in and how this work changed how others understood and valued the innovation and were able to integrate it within existing workflows. CFIR helped us to think about the influence of factors beyond the organizational site level. Combining these approaches provided concrete strategies for healthcare leaders to support ongoing implementation - such as addressing resource constraints and supporting time for sensemaking.

**Conclusion**

Integrating a process theory and a determinant framework informs and enriches understandings of how cognitive and social processes and contextual factors mutually shape and drive implementation efforts. Our case example illustrates how to combine two approaches rigorously through research design, data collection and analysis.

**Acknowledgements**

Support from the Physician Learning Program funded by Alberta Health. The views expressed herein do not necessarily represent the official policy of the Government of Alberta and Alberta Health Services.

### O6 Meaningful Community Engagement through a Co-Created Theory of Change Process to Promote COVID-19 Testing and Vaccine Equity

#### Nicole A. Stadnick^1,2,3*^, Kelli L. Cain^4^, William Oswald^5^, Paul Watson^5^, Marina Castelo^5^, Raphael Logoc^5^, Lawrence Ayers^6^, Linda Salgin^7,8^, Shelia Broyles^9,10^, Louise C. Laurent^6^, Keith Pezzoli^11,12^, Borsika Rabin^2,4+^

##### ^1^University of California San Diego, Department of Psychiatry, San Diego, California, USA; ^2^UC San Diego Altman Clinical and Translational Research Institute Dissemination and Implementation Science Center, San Diego, California, USA; ^3^Child and Adolescent Services Research Center, San Diego, California, USA; ^4^University of California San Diego, Herbert Wertheim School of Public Health and Human Longevity Science, San Diego, California, USA; ^5^The Global Action Research Center, San Diego, California, USA; ^6^University of California San Diego, Department of Obstetrics, Gynecology, and Reproductive Sciences, San Diego, California, USA; ^7^San Ysidro Health, San Diego, California, USA; ^8^San Diego State University/University of California San Diego Joint Doctoral Program in Public Health, San Diego, California, USA; ^9^University of California San Diego, Department of Pediatrics, San Diego, California, USA; ^10^Altman Clinical and Translational Research Institute, Community Engagement Unit, San Diego, California, USA; ^11^University of California San Diego, Department of Urban Studies and Planning, San Diego, California, USA; ^12^University of California San Diego Bioregional Center for Sustainability Science, Planning and Design, San Diego, California, USA

###### **Correspondence:** Nicole A. Stadnick (nstadnic@health.ucsd.edu)

^**+**^ **Contributed equally to this work**

**Background**

The National Institutes of Health (NIH) heavily invested in community engagement research efforts to eliminate COVID-19 disparities in testing, clinical trial participation, access to care, and vaccination. This study reports use of a Theory of Change to engage community members who are from or who support underserved communities in two NIH-funded implementation science projects aimed at promoting equitable access to COVID-19 testing and vaccination for underserved communities.

**Method**

Both projects focused on Latino, Black, and immigrant and refugee communities in South/Central San Diego during December 2020-April 2021. Using a participatory action research design, Community Advisory Boards (CAB) were established for each project. CAB members included community organizers, promotors, healthcare providers and administrators, and public health researchers. The CABs were guided through a six-session Theory of Change, to identify necessary conditions that must exist to reduce COVID-19 disparities along with specified actions to create those conditions and a blueprint for assessing the efficacy of those actions. Each session lasted two hours hosted virtually and augmented by interactive web-based activities. There was a live interpreter to facilitate participation of Spanish-speaking CAB members.

**Results**

A Theory of Change for each project was completed. Nine necessary conditions were identified related to: 1) accessible and available services, 2) culturally and linguistically competent programming, 3) investment in trusted community and faith leaders, 4) social safety nets to provide ancillary services. The CABs operationalized corresponding actions to create these conditions and measures to indicate success in creating these conditions that will be evaluated during upcoming Appreciative Inquiry sessions.

**Conclusion**

A CAB-led Theory of Change process, while resource-intensive, yielded a rich opportunity to engage diverse groups that typically are not invited to inform these processes. Dedicated funding and technical support for community engagement are crucial for successful and sustained implementation of public health interventions.

**Trial registration**

Non applicable.

**Consent to publish**

Informed consent obtained from participants to publish.

### O7 Adapting an implementation strategy to the COVID-19 pandemic context

#### Rodrigo Fernando Presotto, Maria Fernanda Lirani Dos Reis, Sulamita Gonzaga Silva Amorim, Kaleb Eliel Ferreira Leme, Carlos Alberto dos Santos Treichel, Michelle Chanchetti Silva, Livia Pinheiro, Rosana Teresa Onocko Campos

##### Department of Collective Health, State University of Campinas, Campinas-SP, BR

###### **Correspondence:** Rodrigo Fernando Presotto (rfpresotto@gmail.com)

**Background**

In the implementation process is essential to consider the different variables part of the intervention context that influences impacts and results [1]. The training offer is a widely used implementation strategy [2], however, its format needed to be adapted due to the restrictions imposed by the COVID-19 pandemic. This study seeks to analyze the impacts of adapting face-to-face training of health professionals to the online format.

**Method**

The mental health training included 97 professionals in five synchronous meetings lasting 3 hours each, with videos and questions for previous warm-up, small group’s debate, carried out using a videoconference application with the support of a virtual learning management system. At the end, a focus group with stakeholders was carried out, aiming to assess barriers and facilitators of adaptation. A narrative of the material was carried out, analyzed from the Gadamerian hermeneutics framework [3].

**Results**

In the barriers, the “inhibition” for verbal communication was mentioned, the “increased work overload” making participation harder and difficulties with internet connection in “accessibility”. In the facilitators: the “adjustments” of the format allowed the continuity of the implementation, and there may also be a reallocation of the time of the classes from the group's functioning. The “acceptability” of the training was perceived as dependent on the motivation of the participants. During videoconferences, using the chat was evaluated as a favorable form of communication. The use of videos was considered an important strategy for greater participation in the discussions.

**Conclusion**

The training adaptation to the online format was considered adequate, as it allowed the implementation to continue, understanding the real possibilities resulting from the restrictions of the pandemic context. The biggest barrier identified was related to difficulties in accessing the internet, which involves specific problems in countries like Brazil, which can be associated with economic and social difficulties.

**Acknowledgments**

This work was supported by the São Paulo Research Foundation (FAPESP) through grants nº 2018/10366-6 and 2020/14309-7.

**Trial Registration**

Non applicable

**Consent to publish**

Non applicable

**References**

1.Powell, B.J., Waltz, T.J., Chinman, M.J. *et al.* A refined compilation of implementation strategies: results from the Expert Recommendations for Implementing Change (ERIC) project. *Implementation Sci* 10, 21 (2015). 10.1186/s13012-015-0209-1

2.Peters DH, Adam T, Alonge O, Agyepong I, Tran N. Implementation research: what it is and how to do it. BMJ: British Medical Journal. 2013;347:f6753.

3.Gadamer HG. Truth and method: fundamental features of a philosophical hermeneutics. 3. ed. Petrópolis: Voices; 2005.

### O8 Roadmap for evidence-based change in nursing homes: a framework synthesis

#### Geertien C Boersema^1,2^, Magda Mulder^1^, Yvonne Botma^1^

##### ^1^School of Nursing, University of the Free State, Bloemfontein, Free State, South Africa; ^2^Department of Health Studies, University of South Africa, Pretoria, Gauteng, South Africa

###### **Correspondence:** Geertien C Boersema (eboergc@unisa.ac.za, 2018187233@ufs4life.ac.za)

**Background**

The COVID-19 pandemic emphasises the need for effective translation of research evidence into practice at nursing homes. Knowledge translation (KT) and evidence-based practice sustainment are challenged in nursing home contexts [1,2]. A roadmap developed from implementation theories can guide nursing home staff, researchers and implementation support practitioners (ISPs) to embed and sustain evidence-based practice.

**Method**

A roadmap was developed as the first step in a best-fit framework synthesis [3] through:

1. A systematic search for peer-reviewed papers on relevant databases from 1995 to 05/2019 reporting KT theory used in nursing homes
2. Systematic screening of titles, abstract, and full papers resulted in six papers
3. Appraisal of the identified theories’ value using the T-CasT appraisal tool [4]. One framework was excluded. The final frameworks selected:
  Ottawa Model of Research Use
  Quality Enhancement Research Initiative framework
  Promoting Action on Research Implementation in Health Services (and i-PARiHS)
  Champions for Skin Integrity model
  Model for implementing guidelines for person-centred care
4. Deconstruction of the frameworks using structural coding followed by synthesis through code mapping to identify common and unique elements [5]. The Normalisation Process Theory (NPT) [6,7] was also integrated.

**Results**

Figure 1 shows the roadmap consisting of a pre-implementation, implementation, evaluation and sustainment phase including critical strategies e.g. building trust, aligning practice-based knowledge, co-designing and facilitation. Lists of relevant determinants and evaluation outcomes ensued from the analysis.

**Conclusion**

The roadmap can be used to plan or facilitate evidence-based change or to gain insight into existing change processes in nursing homes.

**References**

1. Von Treurer KM, McCabe MP, Karantzas G, Mellor D, Konis A, Davison TE. Facilitating staff adoption of new policies and procedures in aged care through training for readiness for change. J Appl Gerontol. 2020;00:1-8.

2. Pimentel CG, Mills WL, Palmer JA, Dillon K, Sullivan JL, Wewiorski NJ, et al. Blended facilitation as an effective implementation strategy for quality improvement and research in nursing homes. J Nurs Care Qual. 2019;34(3):210-216.

3. Booth A, Carroll C. How to build up the actionable knowledge base: the role of ‘best fit’ framework synthesis for studies of improvement in healthcare. BMJ Qual Saf. 2015;24:700-708.

4. Birken SA, Rohweder CL, Powell BL, Shea CM, Scott J, Leeman J, et al. T-CaST: an implementation theory comparison and selection tool. Implement Sci. 2018;13(143).

5. Saldaña J. The coding manual for qualitative researchers. 4th edition. London: SAGE Publishing; 2021.

6. May C, Finch T, Mair F, Ballini L, Dowrick C, Eccles M, et al. Understanding the implementation of complex interventions in health care: the normalisation process model. BMC Health Serv Res. 2007;7(148):1-7.

7. May C. Towards a general theory of implementation. Implement Sci. 2013;8(18):1-14

**Fig. 1 (abstract O8).**
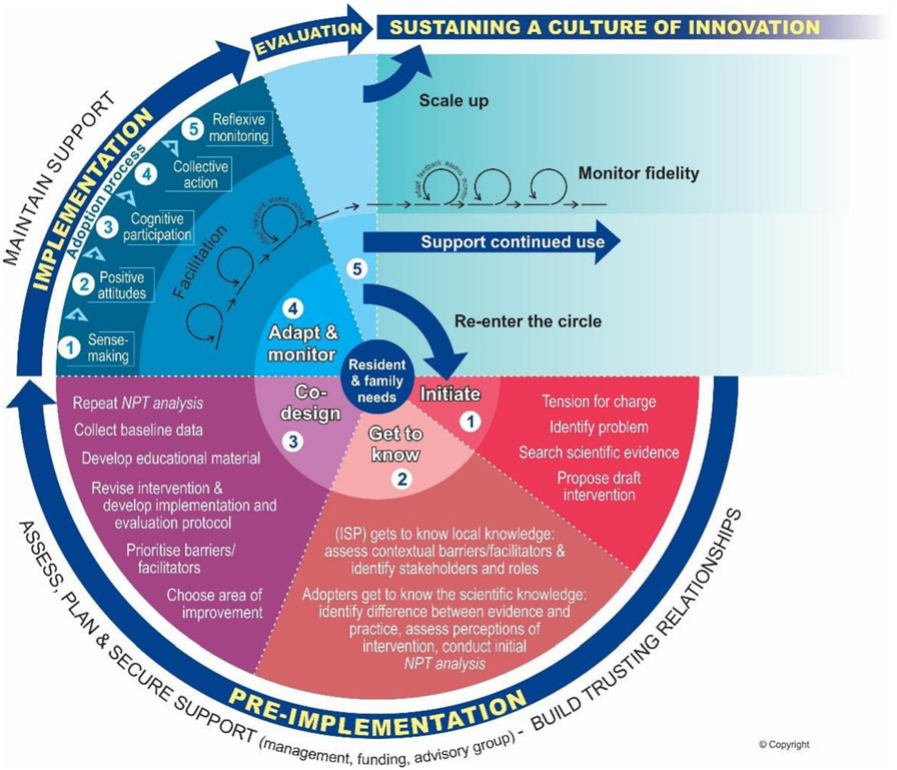
Roadmap for evidence-based change in nursing homes

### O9 Using implementation outcomes and quality measures to evaluate COVIDCare@Home, a Family Medicine Led Remote Monitoring Program for COVID-19 patients

#### Celia Laur^1*^, Payal Agarwal^1,2*^, Kelly Thai^1^, Vanessa Kishimoto^1^, Shawna Kelly^3^, Kyle Liang^1^, R. Sacha Bhatia^5^, Onil Bhattacharyya^1,2^ Danielle Martin^2,3,6,^ Geetha Mukerji^1,4^

##### ^1^Women’s College Hospital Institute for Health Systems Solutions and Virtual Care, University of Toronto, Toronto, ON, Canada; ^2^Department of Family and Community Medicine, University of Toronto, Toronto, ON, Canada; ^3^Women's College Hospital, Toronto, ON, Canada; ^4^Department of Medicine, University of Toronto, Toronto, ON, Canada; ^5^Ontario Health, Toronto, ON, Canada; ^6^Dalla Lana School of Public Health, University of Toronto, Toronto, ON, Canada

###### **Correspondence:** Celia Laur (Celia.Laur@wchospital.ca); Payal Agarwal (payal.agarwal@wchospital.ca)

**Background**

COVIDCare@Home (CC@H) is a multi-faceted, interprofessional team-based remote monitoring program developed at Women’s College Hospital, Toronto, Canada, for newly diagnosed COVID-19 patients in the community. CC@H offers virtual visits to address the clinical and socioeconomic needs of patients during the acute phase of COVID-19. CC@H is designed based on Greenhalgh et al., 2020 [1] remote assessment of patients with COVID-19 in primary care.

**Method**

The multi-method evaluation followed elements of the conceptual framework [2] specifically focused on implementation outcomes and service quality including: feasibility, adoption, safety, effectiveness, equity, and patient-centeredness. These domains were explored using utilization data (EMR data), patient-experience data (a survey and a post-discharge questionnaire), provider-experience data (surveys, interviews and focus groups) and stakeholder perspectives (interviews). Descriptive analysis was conducted for survey results and EMR data. Deductive content analysis was conducted for interviews and focus groups, mapping to implementation and quality domains.

**Results**

In the first 8 months (April – December 2020), 616 patients were enrolled; 55% (n=337) were female, median age of 35 years. 3412 remote visits were conducted, including 149 visits with social workers/mental health professionals. There were 5-visits per patient (median; IQR=4), with a median of 7-days of follow-up (IQR=27), and 3 days between swab result and first visit (median; IQR=3). 60% (n=117) of patients who completed the post-discharge questionnaire (n=194) felt they were discharged at the right time, listing regular check-ins and reassurance as benefits of the program. Interviews with providers and stakeholder indicated that the program rapidly adapted to meet the needs of patients and the healthcare system, providing comprehensive care beyond a COVID-19 diagnosis.

**Conclusion**

CC@H is true evidence into practice as it was designed based on an academic publication to meet a strong health system need. Using a multi-disciplinary approach, the CC@H team remotely supported the clinical and socioeconomical needs of their patients.

**Trial Registration**

Non Applicable

**Consent to publish**

Non Applicable

**References**

1. Greenhalgh T, Koh G C H, Car J. Covid-19: a remote assessment in primary care *BMJ* 2020; 368 :m1182 doi:10.1136/bmj.m1182

2. Proctor E, Silmere H, Raghavan R, et al. Outcomes for implementation research: conceptual distinctions, measurement challenges, and research agenda. *Adm Policy Ment Health*. 2011;38(2):65-76. doi:10.1007/s10488-010-0319-7

### O10 Monitoring and evaluating the impact of a research partnership project on the needs of Canadians with disabilities during and after the COVID-19 pandemic: The COVID-19 Disability Survey

#### Femke Hoekstra^1,2^, Pinder DaSilva^3^, Cameron Gee^2,4^, Tara Joy Knibbe^3^, Meagan O’Neill^3^, Adrienne R. Sinden^1^, Joan Ubeda-Colomer^5^, Kathleen A. Martin Ginis^1,2,6,7^

##### ^1^School of Health and Exercise Sciences, University of British Columbia, Kelowna, BC, Canada; ^2^International Collaboration on Repair Discoveries, University of British Columbia, Vancouver, BC, Canada; ^3^Abilities Centre, Whitby, ON, Canada; ^4^Department of Orthopaedics, Faculty of Medicine, University of British Columbia, Vancouver, BC, Canada; ^5^Departament d'Educació Física i Esportiva, University of Valencia, Valencia, Spain; ^6^Centre for Chronic Disease Prevention and Management, Southern Medical Program, University of British Columbia, Kelowna, BC, Canada; ^7^Division of Physical Medicine & Rehabilitation, Faculty of Medicine, University of British Columbia, Vancouver, BC, Canada

###### **Correspondence:** Femke Hoekstra (femke.hoekstra@ubc.ca)

**Background**

The generally slow process of translating research findings to community and policy settings is unacceptable during a pandemic. Research partnerships, in which researchers and research users work together to conduct and disseminate research, have the potential to accelerate and improve knowledge translation processes. This study aims to monitor and evaluate the impact of a research partnership focusing on the needs of Canadians with disabilities during and after the COVID-19 pandemic.

**Method**

The COVID-19 Disability Survey is a research partnership project led by Abilities Centre and the Canadian Disability Participation Project in consultation with four community organizations [1]. The survey, available in English, French, plain language, and American Sign Language, was developed to collect information on experiences, needs, and concerns of Canadians with disabilities during and after the pandemic. The survey is promoted via partners and social media. Reports outlining key findings are published in accessible formats (**Figure 1**). Monitoring and evaluation of the uptake are ongoing [2].

**Results**

713 and 443 participants completed the survey, respectively, between Jun-Dec ‘20 and Dec ‘20–April ‘21. Promotion and dissemination activities included >60 social media posts, a press release, five government/community presentations, and three open-access reports. Findings have supported policy change in Ontario regarding access to physical activity programs.

**Conclusion**

This study illustrates a unique research partnership initiative, in which real-time information is being collected and disseminated in an inclusive and accessible manner, to help support communities and governments ensure that COVID-19 response strategies meet the needs of people of all abilities.

**References**

1. Hoekstra, F, Martin Ginis, K.A., Sinden, A, et al. COVID-19 Disability Survey. (2021). 10.17605/OSF.IO/Z4GR2

2. Hoekstra, F., Martin Ginis, K.A., Allan, V. et al. Evaluating the impact of a network of research partnerships: a longitudinal multiple case study protocol. *Health Res Policy Sys* 16, 107 (2018). 10.1186/s12961-018-0377-y

**Fig. 1 (abstract O10).**
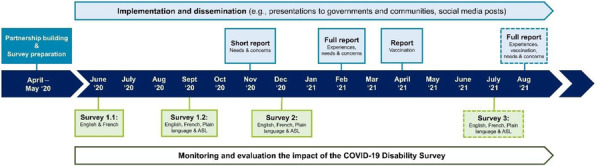
Overview of the key activities of the COVID-19 Disability Survey Project (www.disabilitysurvey.ca). The project will continue after August 2021 to record changes during the COVID-19 recovery period. Frequencies of new surveys and reports will be determined based on changes in COVID-19 related regulations and milestones

### O11 The importance of implementation strategies for successful implementation of evidence-based interventions

#### Lisa R Hirschhorn^1^, Amelia VanderZanden^2^, Thomas Jovial Ntawukuriryayo^2^, Kedest Mathewos^2^, Alemayehu Amberbir^2^, Raj Kumar Subedi^3^, Felix Sayinzoga^4^, Fauzia Akhter Huda^5^, Agnes Binagwaho^2^

##### ^1^Feinberg School of Medicine, Northwestern University, Chicago, IL, USA; ^2^University of Global Health Equity, Kigali, Rwanda ^3^ Nepal Public Health Foundation, Kathmandu, Nepal; ^4^Maternal and Child Health Division, Rwanda Biomedical Center, Kigali, Rwanda; ^5^International Centre for Diarrhoeal Disease Research, Bangladesh (icddr,b), Dhaka, Bangladesh

###### **Correspondence:** Lisa R Hirschhorn (lisa.hirschhorn@northwestern.edu)

**Background**

Between 2000 and 2015, countries worldwide worked to make dramatic reductions in the number of child deaths [1–3]. Six “Exemplar” countries, Senegal, Bangladesh, Peru, Nepal, Rwanda, and Ethiopia, achieved greater declines in under-5 mortality rates (U5MR) than geographic and economic peers. We explore implementation strategies shared across countries to implement evidence-based interventions (EBIs) known to reduce U5M, finding strategies generalizable to other contexts for countries working to reduce U5M.

**Method**

We conducted a series of mixed methods case studies of the Exemplar countries using a common methodology based on a new hybrid implementation research framework and theory of change identifying pathways to reducing amenable U5M[4]. We used multiple case studies methodology [5] to explore the countries’ common and unique experiences, prioritizing contextual factors, implementation strategies, and implementation outcomes, across the five stages of EBI implementation: Exploration, Preparation, Implementation, Adaptation, and Sustainment. We present implementation strategies shared across countries.

**Results**

Common implementation strategies across countries included multisectoral collaboration, data use for understanding gaps and decision-making, adaptation during implementation, and community engagement and education (Table 1). Less common but important when effectively implemented were strategies including prioritizing neonatal mortality, and donor and implementing partner coordination.

**Conclusion**

We found that countries successful in dropping U5M had common strategies to implement EBIs active at many levels from national-level policy setting to involvement at the community-level. Adapted for local context, these strategies can be adopted by other countries looking to accelerate work to reduce deaths among children everywhere.

**References**

1. Lozano R, Wang H, Foreman KJ, Rajaratnam JK, Naghavi M, Marcus JR, et al. Progress towards Millennium Development Goals 4 and 5 on maternal and child mortality: An updated systematic analysis. Lancet [Internet]. 2011 [cited 2021 Mar 14];378(9797):1139–65. Available from: https://pubmed.ncbi.nlm.nih.gov/21937100/

2. Gakidou E, Oza S, Fuertes CV, Li AY, Lee DK, Sousa A, et al. Improving child survival through environmental and nutritional interventions: The importance of targeting interventions toward the poor. J Am Med Assoc [Internet]. 2007 Oct 24 [cited 2021 Mar 14];298(16):1876–87. Available from: https://pubmed.ncbi.nlm.nih.gov/17954539/

3. Ruhago GM, Ngalesoni FN, Norheim OF. Addressing inequity to achieve the maternal and child health millennium development goals: Looking beyond averages. BMC Public Health [Internet]. 2012 [cited 2021 Mar 14];12(1). Available from: https://pubmed.ncbi.nlm.nih.gov/23270489/

4. Hirschhorn LR, Frisch M, Ntawukuriryayo JT, VanderZanden A, Donahoe K, Mathewos K, et al. Development and application of a hybrid implementation research framework to understand success in reducing under-5 mortality in Rwanda. Gates Open Res [Internet]. 2021 Mar 29 [cited 2021 Apr 1];5:72. Available from: https://gatesopenresearch.org/articles/5-72/v1

5. Bartlett L, Vavrus F. Rethinking case study research: a comparative approach. [Internet]. New York: Routledge; 2017. 140 p. [cited 2021 Apr 1];Available from: https://www.routledge.com/Rethinking-Case-Study-Research-A-Comparative-Approach/Bartlett-Vavrus/p/book/9781138939523

**Table 1 (abstract O11).**
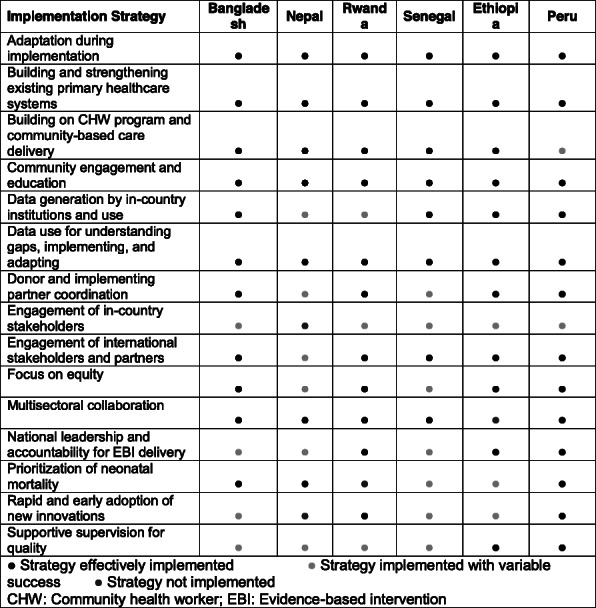
Common implementation strategies in the reduction of amenable under-5 mortality through health system-based evidence-based interventions

### O12 Using the Context and Implementation of Complex Interventions (CICI) framework to identify contextual determinants of people-centred tuberculosis care provision in South Africa: methodological insights from a theory‑generating case study

#### Jamie Murdoch^1^, Robyn Curran^2^, André van Rensburg^3^, Max Bachmann^4^, Ajibola Awotiwon^2^, Nadine Seward^5^, Inge Petersen^3^, Lara Fairall^2,6^

##### ^1^School of Health Sciences, University of East Anglia, NR4 7TJ, Norwich, UK; ^*2*^Knowledge Translation Unit, University of Cape Town Lung Institute and Department of Medicine, University of Cape Town, South Africa; ^3^Centre for Rural Health, University of KwaZulu-Natal, South Africa; ^4^Department of Population Health and Primary Care, Norwich Medical School, University of East Anglia, Norwich, UK; ^*5*^Centre for Implementation Science, Health Service and Population Research Department, Institute of Psychiatry, Psychology & Neuroscience, King’s College London, UK; ^6^King’s Global Health Institute, King’s College London, UK

###### **Correspondence:** Jamie Murdoch (jamie.murdoch@uea.ac.uk)

**Background**

Determinant frameworks provide a means for thinking about and investigating context so as to inform the design and evaluation of implementation programmes. We used the Context and Implementation of Complex Interventions (CICI) framework [1] to investigate the contextual determinants of providing high-quality people-centred TB care in one district in South Africa. Findings were used to inform intervention development to reduce TB deaths and incidence, forming part of the HeAlth System StrEngThening in Sub-Saharan Africa (ASSET) research programme.

**Method**

We applied a theory-building case study design using the CICI framework. Between February and November 2019, we used mixed methods in six public-sector primary healthcare facilities and one public-sector hospital serving impoverished urban and rural communities in the Amajuba district of KwaZulu-Natal province, South Africa. Qualitative data included stakeholder interviews (TB service users, health workers, community health workers, managers), observations and documentary analysis. Quantitative data included routine data on sputum testing and TB deaths. Data were inductively analysed and mapped onto the seven CICI contextual domains.

**Results**

Delayed diagnosis, limited psychosocial support for patients and staff, patients lost to follow-up and inadequate infection control were caused by an interaction between multiple contextual determinants and domains [2]. An additional domain was added to the CICI framework to incorporate many features of TB care we identified within healthcare facilities. Mapping findings onto domains proved challenging as multiple domains were often applicable to a single determinant. This process also did not facilitate analysis of interactions between different determinants to produce the problems we observed.

**Conclusion**

Frameworks such as CICI provide a useful organising structure to identify and evaluate contextual determinants. Caution is required when translating theoretical constructs of context into methods and analytical techniques. Researchers need to ensure contextual determinants are not artificially demarcated and nuanced interactions between determinants are captured empirically.

**Trial Registration**

Non applicable

**Consent to publish**

Yes

**References**

1. Pfadenhauer LM, Gerhardus A, Mozygemba K, Lysdahl KB, Booth A, Hofmann B, et al. Making sense of complexity in context and implementation: the Context and Implementation of Complex Interventions (CICI) framework. Implement Sci. 2017;12(1):21.

2. Murdoch, J., Curran, R., van Rensburg, A.J. et al. Identifying contextual determinants of problems in tuberculosis care provision in South Africa: a theory-generating case study. Infect Dis Poverty. 2021; 10:67 10.1186/s40249-021-00840-5

### O13 Operationalising rapid implementation – Lessons from Academic Health Science Network experience during the COVID-19 pandemic in England

#### Alexandra Ziemann^1^, Andrew Sibley^2^, Sam Tuvey^3^, Harry Scarbrough^1,4^, Sarah Robens^3^

##### ^1^Centre for Healthcare Innovation Research, City, University of London, London, EC1V 0HB, UK; ^2^Wessex Academic Health Science Network, Southampton, Hampshire, SO16 7NP, UK; ^3^South West Academic Health Science Network, Exeter, Devon, EX2 5FD, UK; ^4^The Business School, City, University of London, London, EC1V 0HB, UK

###### **Correspondence:** Alexandra Ziemann (alexandra.ziemann@city.ac.uk)

**Background**

Rapid approaches seem particularly pertinent in implementation science to reach the field’s underlying goal of closing the know-do gap. Smith et al. recently conceptualised rapid implementation as achieving “speed and efficiency, by redefining rigour, and adapting both methods [and] design” [1, p. 9]. The COVID-19 pandemic offered a “natural laboratory” to learn about rapid implementation. Our aim was to explore how rapid implementation was operationalised by Academic Health Science Networks (AHSN) in England during the first wave of the COVID-19 pandemic.

**Method**

We organised three 90-minute, online, semi-structured focus groups with 26 operational and senior managerial staff from 14 of the 15 AHSNs in June-July 2020. Participants were recruited purposefully and on a voluntary basis. Participants presented a case study about their approaches to implementing innovations between March-June 2020 and discussed their experiences and lessons learned. The focus groups were audio-recorded, transcribed verbatim and analysed using qualitative thematic analysis following a grounded theory approach.

**Results**

AHSNs increased the pace of their innovation implementation work to support the response to COVID-19, e.g., remote consultations. AHSNs operationalised rapid implementation by: 1) Accelerating existing innovations and building on existing relationships/networks; 2) Using remote working for more efficient stakeholder engagement, training, and dissemination; 3) More agile working and adaption of innovations to meet changing local needs/contexts, 4) Using emergent enablers, e.g. stakeholder/policymaker mindsets accepting lower rigour/quality, identifying a common purpose, and allowing for the generation of new evidence from evaluations of rapid implementation.

**Conclusion**

A combination of remote and agile ways of working and a new enabling context allowed for more rapid implementation of innovations during the COVID-19 pandemic. Key approaches to be taken forward could be the progressively proven remote ways of working and the increased focus on adaptive strategies to increase implementation efficiency and pace after COVID-19.

**Trial Registration**

NA

**Consent to publish**

If applicable, please see guidelines

**Reference**

1. Smith J, Rapport F, O’Brien TA, Smith S, Tyrrell VJ, Mould EV, Long JC, Gul H, Cullis J, Braithwaite J. The rise of rapid implementation: a worked example of solving an existing problem with a new method by combining concept analysis with a systematic integrative review. BMC Health Serv Res. 2020;20:1-4.

### O14 Tailored implementation of internet based cognitive behavioural therapy (ImpleMentAll): Process evaluation of the ItFits-toolkit

#### Tracy Finch^1^, Sebastian Potthoff^2^, Carl May^3^, Melissa Girling^1^, Neil Perkins^2^, Christiaan Vis^4^, Josien Schuurmans^6^, Leah Bührmann^4^, Anne Etzelmueller^5^, Claire van Genugten^6^, Tim Rapley^2^

##### ^1^Department of Nursing, Midwifery & Health, Northumbria University, Newcastle upon Tyne, NE7 7XA, UK; ^2^Department of Social Work, Education & Community Wellbeing, Northumbria University, Newcastle upon Tyne, NE7 7XA, UK’; ^3^Faculty of Public Health and Policy, London School of Hygiene and Tropical Medicine, London, WC1H 9SH, UK; ^4^Department of Clinical, Neuro-, & Developmental Psychology, Faculty of Behavioural and Movement Sciences, VU Amsterdam, Amsterdam, The Netherlands; ^5^HelloBetter/GET.ON Institute, Hamburg, 20249, Germany; ^6^Department of Research and Innovation, GGZ inGeest, Specialized Mental Health Care, Amsterdam, 1081HJ, The Netherlands

###### **Correspondence:** Tracy Finch (tracy.finch@northumbria.ac.uk)

**Background**

The ImpleMentAll study [1] developed the ItFits-toolkit, a self-guided online platform to facilitate implementation of tailored strategies for internet-based cognitive behavioural therapy (iCBT) services. Informed by Implementation Science, users progress through four modules covering work on barriers, strategies, planning, project execution and review. The effectiveness trial reported a small but significant positive effect of ItFits-toolkit on the normalisation of iCBT services [2]. An embedded process evaluation explored how teams engaged with the toolkit across different settings.

**Method**

Thirteen sites participated across nine countries (Europe and Australia). Qualitative data included remote interviews (n=55) spanning multiple time-points. Interview participants (n=30) included implementation leads (n=19), implementation team members (n=9), and other stakeholders (n=2). Observations from support calls (n=19) between trial team and implementation teams were also included. Descriptive data regarding goals, barriers, strategies and implementation plans were collected through the digital platform. Qualitative data were analysed thematically, using a team-based approach.

**Results**

Twenty implementation projects (in 10/13 sites) reached an adequate level of completion. Implementation teams engaged well with the overall logic and core elements (guidance and resources) of ItFits-toolkit. The toolkit facilitated more structured and purposive engagement with stakeholders. The teams made the toolkit ‘at home’, adapting how they worked with it within their existing organisational norms, logics and routines. Toolkit implementation was affected by pre-existing and emerging implementation interests, positions, or ideas of teams and other stakeholders involved in the projects. Project progress was affected by internal and external issues and events (eg. Covid-19); and the trial context itself.

**Conclusion**

ItFits-toolkit changed the way implementation teams worked to develop and implement tailored strategies. Implementation work became more focused and involved engaging a broader range of stakeholders. However achieving implementation objectives depends on capacity to address multiple internal and external challenges to this work, and on adequate timelines for assessing impact.

**Trial Registration**

ClinicalTrials.gov NCT03652883. Retrospectively registered on 29 August 2018

**Acknowledgements**

Authored on behalf of the ImpleMentAll study consortium.

**References**

1. Bührmann, L., Schuurmans, J., Ruwaard, J. *et al.* Tailored implementation of internet-based cognitive behavioural therapy in the multinational context of the ImpleMentAll project: a study protocol for a stepped wedge cluster randomized trial. *Trials* **21,** 893 (2020). [cited 2021 March 31]. Available from: 10.1186/s13063-020-04686-4

2. Effectiveness of the ItFits-toolkit. Christiaan Vis On behalf of the ImpleMentAll Effectiveness Study team (WP3):Josien Schuurmans, Claire van Genugten, Adriaan Hoogedoorn. ImpleMentAll Final Conference [online], 16^th^-17^th^ March 2021. [cited 2021 March 31] available fromg https://www.implementall.eu/9-outcomes-and-resources.html

### O15 Understanding geographic equity in the coverage of facility-based delivery in six Exemplar countries

#### Jovial Thomas Ntawukuriryayo^1^, Amelia VanderZanden^1^, Kedest Mathewos^1^, Alemayehu Amberbir^1^, Lisa R. Hirschhorn^1,2^, Agnes Binagwaho^1^

##### ^*1*^University of Global Health Equity, Kigali, 6955, Rwanda; ^2^Department of Medical Social Sciences, Northwestern University Feinberg School of Medicine, Chicago, IL, 60611, USA

###### **Correspondence:** Jovial Thomas Ntawukuriryayo (tntawukuriryayo@ughe.org)

**Background**

Compared to regional and socioeconomic peers, Senegal, Ethiopia, Rwanda, Peru, Nepal, and Bangladesh were “Exemplar” countries for successfully reducing under-5 mortality (U5M) nationally, through implementation of evidence-based interventions (EBIs) known to reduce U5M [1-6]. However, high national EBI coverage does not always mean equitable coverage across the country [7]. We completed implementation research case studies to understand national and subnational EBI implementation [1-6]. We describe results for facility-based delivery (FBD), critical for reducing neonatal and maternal mortality and requiring a trusted health system.

**Method**

We used subnational data from demographic and health survey reports for the six countries, to compare subnational FBD coverage for/or around 2000 and 2015. We calculated absolute equity gaps (difference between the highest and lowest subnational EBI coverage) for each country. Factors and implementation strategies that influenced geographic equity in FBD coverage were identified from the case studies [1-6].

**Results**

For 2000-2015, the absolute geographic equity gap in FBD coverage varied within the countries, with a decline in three, ranging from a slight decrease (Senegal) to an extensive reduction (Rwanda), with gap increasing in the rest (table 1). Contextual barriers included geographic obstacles and cultural beliefs. Effective strategies included free FBD, cultural adaptations, health system strengthening, and leveraging existing community health programs.

**Conclusion**

The changing geographic equity gap in FBD coverage varied within countries who were leaders in U5M reduction. Implementation science methods can identify potential barriers and strategies to address subnational inequity in EBI delivery.

**Trial Registration**

NA

**Consent to publish**

NA

**References**

1. Binagwaho A, Udoh K, Donahoe K, Gasanova Z, Sall M, Hirschhorn LR. Exemplars in Under-5 Mortality: Senegal Case Study [Internet]. 2018 [cited 2021 May 13]. Available from: https://www.exemplars.health/-/media/files/egh/resources/underfive-mortality/senegal/senegal-case-study-_-final-28082020.pdf

2. Hirschhorn LR, Sayinzoga F, Beyer C, Donahoe K, Binagwaho A. Exemplars in Under-5 Mortality: Rwanda Case Study [Internet]. 2018 [cited 2021 May 13]. Available from: https://www.exemplars.health/-/media/files/egh/resources/underfive-mortality/rwanda/rwanda-case-study_-final-28082020.pdf

3. Hirschhorn LR, Garcia PJ, Larson A, Drown L, Carcamo MH, Frisch M, et al. Exemplars in Under-5 Mortality: Peru Case Study [Internet]. 2020 [cited 2021 May 13]. Available from: https://www.exemplars.health/-/media/files/egh/resources/underfive-mortality/peru/peru-case-study-_-final-28082020.pdf

4. Hirschhorn LR, Subedi RK, Beyer C, Adhikari K, Bastola S, Donahoe K, et al. Exemplars in Under-5 Mortality: Nepal Case Study [Internet]. 2018 [cited 2021 May 13]. Available from: https://www.exemplars.health/-/media/files/egh/resources/underfive-mortality/nepal/nepal-case-study_-final-_10042020.pdf

5. Binagwaho A, Teklu AM, Drown L, Udoh K, Frisch M, Ntawukuriryayo JT, et al. Exemplars in Under-5 Mortality: Ethiopia Case Study [Internet]. 2020 [cited 2021 May 13]. Available from: https://www.exemplars.health/-/media/files/egh/resources/underfive-mortality/ethiopia/ethiopia-case-study-_-final-_10042020.pdf

6. Binagwaho A, Udoh K, Ntawukuriryayo T, Faruk O, Mahmood HR, Huda FA, et al. Exemplars in U5M: Bangladesh Case Study [Internet]. 2019 [cited 2021 May 13]. Available from: https://www.exemplars.health/-/media/files/egh/resources/underfive-mortality/bangladesh/bangladesh-case-study-_-final-28082020.pdf

7. WHO. Immunization Analysis and Insights: Subnational immunization coverage data [Internet]. World Health Organization. 2021 [cited 2021 Nov 3]. Available from: https://www.who.int/teams/immunization-vaccines-and-biologicals/immunization-analysis-and-insights/global-monitoring/immunization-coverage/subnational-immunization-coverage-data

8. NISR, MINECOFIN, MOH, ICF International. Rwanda Demographic and Health Survey (DHS) 2014-15 [Internet]. Kigali, Rwanda; Rockville, Maryland, USA; 2016 [cited 2020 Feb 26]. Available from: https://dhsprogram.com/pubs/pdf/FR316/FR316.pdf

9. ONP, ORC Macro. Enquête Démographique et de Santé (DHS), Rwanda 2000 [Internet]. Kigali, Rwanda; Calverton, Maryland, USA; 2001 [cited 2020 Feb 26]. Available from: https://dhsprogram.com/publications/publication-fr125-dhs-final-reports.cfm

10. INEI, USAID, UNICEF. Peru Encuesta Demográfica y de Salud Familiar 2000 [Internet]. Lima, Peru; 2001 [cited 2020 Feb 21]. Available from: https://dhsprogram.com/pubs/pdf/FR120/FR120.pdf

11. INEI. Peru Encuesta Demográfica y de Salud Familiar (ENDES) 2014 [Internet]. Lima, Peru; 2015 [cited 2020 Feb 21]. Available from: https://dhsprogram.com/pubs/pdf/FR310/FR310.pdf

12. Mitra S, Al-Sabir A, Saha T, Kumar S. Bangladesh Demographic and Health Survey 1999-2000 [Internet]. Dhaka, Bangladesh; Calverton, Maryland; 2001 [cited 2019 Sep 14]. Available from: https://dhsprogram.com/pubs/pdf/FR119/FR119.pdf

13. NIPORT, ACPR II. Bangladesh Health Facility Survey 2014 [Internet]. Dhaka, Bangladesh; 2016 [cited 2019 Sep 13]. Available from: https://dhsprogram.com/pubs/pdf/SPA23/SPA23.pdf

14. MOH, New ERA, ICF. Nepal Demographic and Health Survey 2016 [Internet]. 2016 [cited 2020 Feb 26]. Available from: https://www.dhsprogram.com/pubs/pdf/fr336/fr336.pdf

15. MOH, ERA N, Macro O. Nepal Dermographic and Health Survey [Internet]. Vol. 8, Family Health Division, Ministry of Health, New ERA, and ORC Macro. 2002. Available from: https://dhsprogram.com/pubs/pdf/FR132/FR132.pdf

**Table 1 (abstract O15).** Subnational equity data for FBD for Ethiopia, Peru, Bangladesh, Nepal, Rwanda, and Senegal [8-15]

Evidence-Based Interventions (EBIs)	Equity	Ethiopia		Peru		Bangladesh		Nepal		Rwanda		Senegal	
		2000	2016	2000	2014	1999	2014	2001	2016	2000	2014	2005	2016
**Facility-based delivery (%)**	National coverage	5	33	58	89	8	38	9	57	26	91	62	74
	Region with highest coverage	67	97	92	100	14	55	11	66	71	94	85	92
	Region with lowest coverage	3	15	20	67	4	23	4	50	21	89	47	55
	Absolute equity gap	64	82	72	33	10	32	7	16	50	5	38	37
	**Change in absolute equity gap**	**18**		**-39**		**22**		**9**		**-45**		**-1**	
	Note: • Absolute equity gap= Absolute value for a region with the highest coverage - Absolute value for a region with the lowest coverage • Change in absolute equity gap= absolute equity gap in the end year - absolute equity gap in the start year												

### O16 Facilitating the implementation of remote consultations: development of evidence-informed visual guidelines

#### Jordi Piera-Jiménez^1,2,3^, Jesús Berdún^4^, Oscar Solans^2,5^, Anna Forment^6^, Liliana Ramalho^6^, Judit Baiget^6^, Gerard Carot-Sans^1,2^, Pol Pérez Sust^1^

##### ^1^Servei Català de la Salut, Barcelona, Spain; ^2^Digitalization for the Sustainability of the Healthcare System, Sistema de Salut de Catalunya, Barcelona, Spain; ^3^Open Evidence Research Group, Universitat Oberta de Catalunya, Barcelona, Spain; ^4^Fundació TIC Salut i Social, Barcelona, Spain; ^5^Institut Català de la Salut, Barcelona, Spain; ^6^Everis, Barcelona, Spain

###### **Correspondence:** Jordi Piera-Jiménez (jpiera@catsalut.cat)

**Background**

The pandemic caused by the coronavirus disease 2019 has severely disturbed routine clinical practice. To contain the spread of the disease and protect both clinicians and citizens, healthcare facilities closed non-essential consultations. In order to ensure access and care continuity, the Catalan Health Service deployed at-scale two solutions for remote consultations (synchronous and asynchronous) [1]. We sought to facilitate the implementation of remote consultations by developing evidence-informed visual guidelines in line with the previous work conducted by T. Greenhalgh et al [2].

**Method**

Taking advantage of the LATITUD project, we convened a multidisciplinary task force (including practitioners, scholars, and scientific societies) aimed at developing the guidelines throughout a participatory co-design process. The work was conducted between May and June 2020 in three steps: 1) Evidence collection, based on a systematic search of empirical qualitative studies and grey literature, 2) Data processing, in which consolidated evidence was broke down into six areas of interest (use case scenarios, organization and expertise, tools and requirements, legal aspects and ethics, good practices and checklists), and 3) Guidelines development, in which the consecutive versions of the guidelines produced by an editorial group were validated by the taskforce.

**Results**

The group produced two pairs of infographics. Each pair provided a specific implementation guideline for healthcare service leaders and healthcare professionals. The first one focused on primary care [3] and the second one on specialized care centers, intermediate and long-term care centers, and mental health and addiction centers [4].

**Conclusion**

In the challenging context of system overburden and urgent need for rapid implementation of remote consultations, which precludes setting up facilitator teams, providing infographic guidelines may be a key facilitator in the implementation strategy. We believe the guidelines targeting implementation leads may have enhanced the uptake of remote consultations across the Catalan health system.

**Acknowledgements**

The LATITUD project was co-funded by the European Union via the Structural Reform Support Programme and implemented in cooperation with the European Commission/European Commission’s Directorate General for Reform Support (DG REFORM).

This work is being presented on behalf of the co-design group members, without whom this work would not have been possible.

**Consent to publish**

All participants consented for publication.

**References**

1. Pérez Sust P, Solans O, Fajardo JC, Medina Peralta M, Rodenas P, Gabaldà J, et al. Turning the Crisis Into an Opportunity: Digital Health Strategies Deployed During the COVID-19 Outbreak. JMIR Public Heal Surveill [Internet]. 2020;6(2):e19106. Available from: http://publichealth.jmir.org/2020/2/e19106/

2. Greenhalgh T, Koh GCH, Car J. Covid-19: a remote assessment in primary care. BMJ [Internet]. 2020;368. Available from: [https://www.bmj.com/content/368/bmj.m1182

3. Use of Non-Face-to-face Care channels in primary care [https://ticsalutsocial.cat/en-use-of-non-face-to-face-care-channels-in-primary-care/]

4. Use of Non-Face-to-Face channels in hospital outpatient clinics, community health and mental health centres [https://ticsalutsocial.cat/en-use-of-non-face-to-face-hospital-outpatient-clinics-community-health-and-mental-health-centres/]

### O17 Advocating an agency-structure approach to examine implementation of a compassionate care initiative in mental health settings during Covid-19 in the UK

#### Cindy Brooks^1^, Jackie Bridges^2^, Jane Frankland^2^, Caitlin Burchett^3^, Michelle Myall^1^

##### ^1^NIHR ARC Wessex, University of Southampton, UK; ^2^School of Health Sciences, University of Southampton, UK; ^3^Solent NHS Trust, Hampshire, UK

###### **Correspondence:** Cindy Brooks (C.F.Brooks@soton.ac.uk)

**Background**

This paper advocates how adopting an agency-structure approach enhances understanding of the complexities of implementing a compassionate care initiative (CCI) in two mental health settings during Covid-19 in the UK. Earlier studies reporting on the implementation of CCI in acute hospital settings, whilst identifying improvements in the ability of nurses to provide compassionate care, acknowledged barriers to sustaining implementation including: staffing levels; working priorities; staff priorities and manager’s support [1,2,3]. However, there is limited knowledge of the complex mechanisms involved in the process of implementation of CCI over time and in different settings. To address these limitations, this paper reports on a study adopting an agency-structure approach to examine sustainability of CCI with consideration of a variety of contexts.

**Method**

The study adopts a longitudinal mixed methods case study design [4] in two NHS mental health settings in the UK, involving semi-structured interviews, a staff wellbeing survey with all staff involved in the implementation of CCI (managers, facilitators and frontline care staff including registered nurses) and documentary analysis of key documents. An agency-structure approach using a combination of Structuration Theory (ST) and Normalisation Process Theory (NPT) inform analysis [5,6,7].

**Results**

Emergent findings suggest implementation of CCI during Covid-19 requires a flexible nuanced agency-structure approach to examine the continual interplay between agency and multiple contexts [8], relating to three interconnected spheres:

i. individual (how individuals enact change within an organisation; their professional role, relations with colleagues/patients)
ii. organisational, environmental and cultural factors (e.g. implementation associated activities, organisational infection control guidelines, working patterns)
iii. broader historical, economic and political factors (e.g. policies relating to mental health, compassionate care, self-isolation)

**Conclusion**

Adopting an agency-structure approach affords valuable insight into the complexities of implementation of CCI during Covid-19, as well as flexibility to explore sustainability, given uncertainties surrounding the impact of Covid-19.

**References**

1. Bridges, J, Fuller, A. Creating Learning Environments for Compassionate Care (CLECC): a programme to promote compassionate care by health and social care teams. Int J Older People Nurs. 2015;10(1):48-58.

2. Bridges, J, May C.R., Fuller, A., et al. Optimising impact and sustainability: a qualitative process evaluation of a complex intervention targeted at compassionate care. BMJ Quality & Safety. 2017;26(12):970-77.

3. Bridges, J, Lee, K, Griffiths, P, et al. Creating Learning Environments for Compassionate Care (CLECC): The implementation and evaluation of a sustainable team-based workplace learning intervention. Southampton: University of Southampton; 2019.

4. Yin, R.K. Case study research and applications: design and methods. Thousand Oaks; 2018.

5. May, C and Finch, T. Implementing, embedding, and integrating practices: an outline of normalization process theory. Sociology. 2009; 43(3):535-54.

6. Giddens, A. The constitution of society: Outline of the theory of structuration. Cambridge: Polity Press; 1984.

7. Stones, R. Structuration Theory. Basingstoke: Palgrave-Macmillan; 2005.

8. Myall, M., May, C., Richardson, A., Bogle, S., Campling, N., Dace, S., & Lund, S. Creating pre-conditions for change in clinical practice: the influence of interactions between multiple contexts and human agency. Journal of Health Organization and Management. 2020; Available from: 10.1108/JHOM-06-2020-0240.

### O18 Implementation of an electronic clinical decision support system (eCDSS) for the early recognition and management of diabetes and dysglycaemia in secondary mental healthcare: Protocol for a pilot two-arm randomized controlled cluster trial

#### Dipen Patel^1,2^, Fiona Gaughran^1,2^, Richard Dobson^3^, Tao Wang^3^, Yamiko Msosa^3^, Angus Roberts^3^, Julie Williams^4^, Omar Mustafa^5^

##### ^1^Department of Psychosis Studies, Institute of Psychiatry, Psychology and Neuroscience, King's College London, London, UK; ^2^South London and Maudsley NHS Foundation Trust , London, UK; ^3^Department of Biostatistics & Health Informatics, Institute of Psychiatry, Psychology and Neuroscience, King's College London, London , UK; ^4^Centre for Implementation Science, Health Service and Population Research Department, King's College London, London, UK; ^5^King's College Hospital NHS Foundation Trust, London, UK

###### **Correspondence:** Dipen Patel (Dipen.1.patel@kcl.ac.uk)

**Background**

Severe mental illnesses (SMI), including schizophrenia, bipolar disorder and major depressive disorder are associated with physical health comorbidities and premature mortality. Digital technologies such as electronic clinical decision supports systems (eCDSS) could play a crucial role in improving clinician led management of physical health conditions such as diabetes in SMI.

CogStack@Maudsley is a real-time eCDSS developed to automatically alert clinicians of patients in secondary mental healthcare, with trust approved guideline-based recommendations for diabetes monitoring and management, tailored to the individual patient.

This protocol describes a feasibility study of the implementation of an eCDSS in a mental health inpatient setting to improve clinician led diabetes care.

**Method**

This will be a feasibility study of a two-arm randomized controlled cluster trial conducted in an inpatient mental health trust. Wards will be the unit of recruitment and assigned to either the intervention or control group in a 1:1 ratio, to receive access to the eCDSS or to follow usual care processes and we aim to recruit 4 wards, with a 4 month follow up.

**Results**

We will measure feasibility and acceptability of the eCDSS to clinicians as primary outcomes, alongside secondary outcomes relating to process of care measures such as diabetes screening rates. An evaluation of the implementation of the eCDSS will be conducted. Several implementation outcomes will be evaluated, based on established implementation science frameworks.

**Conclusion**

eCDSS has the potential to improve clinician led management of diabetes in inpatient mental health settings. If found to be feasible and acceptable, then in combination with results of the implementation evaluation, the system can be refined and potential problems with future successful implementation addressed. A larger and more definitive effectiveness trial can then be conducted to assess impact on clinical outcomes and to inform scalability and application to other conditions, in wider mental healthcare settings.

**Trial Registration**

ClinicalTrials.gov, registration no: NCT04792268

### O19 Evaluating the application of the RE-AIM framework: An updated systematic review and exploration of pragmatic application

#### Danielle D’Lima^1^, Tayana Soukup^2^, Louise Hull^2^

##### ^1^Centre for Behaviour Change, Department of Clinical, Educational and Health Psychology, University College London, London, UK; ^2^Centre for Implementation Science, Health Service and Population Research Department, King’s College London, London, UK

###### **Correspondence:** Danielle D’Lima (d.dlima@ucl.ac.uk)

**Background**

The importance of using theories, frameworks and models in implementation research and practice is widely recognised. The Reach Effectiveness Adoption Implementation and Maintenance (RE-AIM) framework is one of the most highly used implementation frameworks. We report a systematic review that provides (a) an updated synthesis of RE-AIM use over time (update of review by Gaglio et al, 2013)^[1]^, (b) explores the pragmatic use of RE-AIM, in a sub-set of articles meeting inclusion criteria, and (c) provides an in-depth exploration of the reasoning and justification for full and pragmatic use of RE-AIM, in a sub-set of articles meeting inclusion criteria.

**Method**

We searched MEDLINE (R) and PsycINFO, via the Ovid interface, between January 2011 and December 2017. The search term ‘RE-AIM’ was used to search for relevant articles. Studies that applied RE-AIM as a planning and/or evaluation framework were eligible for inclusion.

**Results**

157 met inclusion criteria, of which 149 reported using RE-AIM as an evaluation framework, 3 as a planning framework and 5 as a planning and evaluation framework. Reach was the most frequently reported RE-AIM dimension followed by adoption, implementation, effectiveness and maintenance. Fifty articles applied RE-AIM pragmatically (i.e., not in its entirety). Within the sub-set analysis (approximately 10% of articles meeting inclusion criteria), 9/15 articles evaluated all RE-AIM dimensions, therefore justifying the rationale for not evaluating RE-AIM dimensions was not applicable. Of the 6/15 articles that did not evaluate one or more RE-AIM dimensions, 5 articles did not justify the rationale for not evaluating RE-AIM dimensions.

**Conclusion**

RE-AIM has gained increased use in recent years and there is evidence that it is being applied pragmatically. However, the rationale for its pragmatic use is often not reported, making it impossible to rule out that key aspects of the framework have not simply been overlooked.

**Trial Registration:**

Non applicable

**Consent to publish**

Yes

**Reference**

1. Gaglio B, Shoup JA, Glasgow RE. The RE-AIM framework: a systematic review of use over time. Am J Public Health. 2013;103(6):e38-46.

### O20 (Re-)conceptualising implementation depth of healthcare innovations – A systematic review and concept analysis

#### Zuhur Balayah^1^, Charitini Stavropoulou^2^, Yaru Chen^3^, Harry Scarbrough^1^, Amit Nigam^1^, Alexandra Ziemann^3^

##### ^1^The Business School, Centre for Healthcare Innovation Research (CHIR), City, University of London, EC1V 0HB, UK; ^2^School of Health Sciences, Centre for Healthcare Innovation Research (CHIR), City, University of London, EC1V 0HB, UK; ^3^Centre for Healthcare Innovation Research (CHIR), City, University of London, EC1V 0HB, UK

###### **Correspondence:** Zuhur Balayah (zuhur.balayah@cass.city.ac.uk)

**Background**

Implementation depth, the extent to which innovations are implemented successfully, is a matter of great interest in healthcare practice. Yet, the way implementation depth is conceptualised varies between different studies, settings and contexts. The aim of this study is to report on the clarification and re-conceptualisation of implementation depth in healthcare, by synthesising the theoretic scientific literature from multiple disciplinary backgrounds.

**Method**

We applied a pragmatic utility concept analysis approach, a meta-analytic and interpretative method aiming at providing new insights of partially mature concepts using literature as data source. We followed the BeHEMoTh (Behaviour or phenomenon of interest, Health context, Exclusions, Models and Theories) approach for systematically searching for and identifying a comprehensive compilation of concepts from the scientific literature. The following databases were searched: Medline, Embase, CINAHL, PsychInfo, Global Health, HMIC, Business Source Complete, and Social Policy and Practice. In addition to handsearching references of selected publications, key textbooks and citation tracking. First order-concepts’ definitions, characteristics/attributes and boundaries/allied concepts were extracted and analysed to derive second-order concepts of implementation depth.

**Results**

We identified 66 publications that met our eligibility criteria. The preliminary results reveal the consolidated conceptualisation of implementation depth encompasses five concepts: low implementation depth (*abandonment*), high implementation depth (assimilation), normalising and sustaining innovation over time (*sustainability*), removal/reduction or substitution of an existing practice (*de-implementation*), and progression of implementation stages (*stickiness of implementation stages*). The second-order concepts of implementation depth clarify a unified structure to conceptualise the dynamic successes and/or failures of implementation efforts.

**Conclusion**

The consolidated framework of implementation depth delineates the type of implementation ‘success’. It offers a useful heuristic for operationalising shallow to deep implementation, that may be better suited for understanding challenges with sustaining, scaling and spreading healthcare innovations.

### O21 Self-monitoring of blood pressure (SMBP) in pregnancy: a national roll-out in the context of a pandemic

#### Hannah Wilson^1^, Katherine Tucker^2^, Layla Lavallee^2^, Lisa Hinton^3^, Richard J McManus^2^, Lucy C Chappell^1^

##### ^1^School of Life Course Sciences, King’s College London, London, UK; ^2^Nuffield Department of Primary Care Health Sciences, University of Oxford, Oxford, UK; ^3^The Healthcare Improvement Studies Institute, University of Cambridge, Cambridge, UK

###### **Correspondence:** Hannah Wilson (Hannah.1.Wilson@kcl.ac.uk)

**Background**

In April 2020, the Royal College of Obstetricians and Gynaecologists (RCOG) published guidance on establishing services so women with pregnancy hypertension could have additional remote monitoring during the COVID-19 pandemic [1]. To support implementation, NHS England distributed over 16,000 blood pressure (BP) monitors free of charge to maternity providers on request.

**Method**

The evaluation included the following:

1. Survey of 127 maternity providers in England about their implementation of SMBP
2. Survey of 166 women who were currently pregnant or who had had a baby since March 2020 regarding their experiences with SMBP

**Results**

Of 127 providers contacted, 35% responded, of whom most (78%) did not regularly provide BP monitors to pregnant women prior to the COVID-19 pandemic. SMBP was most commonly offered to women who had developed gestational hypertension (89%) and used for additional monitoring (93%) rather than as a replacement for a routine face-to-face contact. Almost all (98%) providers provided written information to women alongside the BP monitor, as provided in the RCOG COVID-19 SMBP guidance. Overall providers were positive about the ability of SMBP to reduce face-to-face contacts (80%). Providers aimed to recycle monitors for multiple women but return rates averaged around 40%. Monitoring was largely undertaken at the request of healthcare professionals (86%). Feedback was strong with 96% feeling safe undertaking SMBP during the COVID-19 pandemic, 78% saying that SMBP made them feel more confident, and 25% more anxious. The most positive aspect reported by women was greater control/insight into their own BP.

**Conclusion**

Many providers in England have commenced a SMBP service since March 2020 to provide additional monitoring in pregnancy. Overall providers and women were positive about use. Consideration needs to be given to the longer term role of SMBP in pregnancy in light of forthcoming trial results, strategies for BP monitor provision and any service reconfigurations post-pandemic.

**Trial Registration**

NA

**Consent to publish**

NA

**Reference**

1. Royal College of Obstetricians and Gynaecologists (RCOG) (2020) Self-monitoring of blood pressure in pregnancy. Accessed online: https://www.rcog.org.uk/globalassets/documents/guidelines/2020-03-30-self-monitoring-of-blood-pressure-in-pregnancy.pdf on 13 May 2020.

### O22 Supporting the physical health of people admitted to mental health wards during the Covid19 pandemic: Prospective implementation evaluation of two novel service developments

#### Ray McGrath^1^, Julie Williams^2^, Isabel McMullen^1^, Prashanth Reddy^3^, Gavin Shields^3^, Fiona Gaughran^4^, Ioannis Bakolis^5^, Andy Healey^6^, Amy Clark^6^, Euan Sadler^7^, Zarnie Khadjesari^8^, Nick Sevdalis^2^ on behalf of the IMPHS study group

##### ^1^South London and Maudsley NHS Foundation Trust, London, SE5 8AZ, UK; ^2^Centre for Implementation Science, King’s College London, London, SE5 8AF, UK; ^3^King’s College Hospital, London, UK; ^4^Psychosis Studies, King's College London, London, SE5 8AF, UK; ^5^Department of Biostatistics and Health Informatics, King’s College London, London, SE5 8AF, UK; ^6^Kings Health Economics, King's College London, London, SE5 8AF, UK; ^7^Department of Nursing, Midwifery and Health, University of Southampton, Southampton, UK; ^8^Behavioural and Implementation Science (BIS) research group, University of East Anglia, Norwich, UK

###### **Correspondence:** Ray McGrath (raymond.mcgrath@slam.nhs.uk)

**Background**

The Covid-19 pandemic emphasised the need to provide robust support to people who are inpatients in psychiatric hospitals. Within the South London and Maudsley NHS Foundation Trust (SLaM) in London, UK, rapid implementation of two initiatives has taken place. Consultant Connect (CC), a clinician to clinician telephone advice service, and the Virtual Physical Health Clinic (VPHC), a virtual clinic staffed by a Consultant Physician and Advanced Clinical Practitioner were launched in June 2020 to support the physical health of inpatients. We report interim data from the prospective evaluation of these interventions, including the uptake and reach of each service and the benefits reported by staff.

**Method**

We are evaluating the implementation process of both services using quantitative data on uptake and reach, and data collected from interviews with clinical staff and through validated implementation outcome assessment measures. We are assessing implementation strategies using the Expert Recommendations for Implementing Change (ERIC) strategies as a framework. We will assess the health economic impact of both services using established health economic methods including cost comparison scenarios and health service utilisation analyses.

**Results**

From June 2020 until April 2021, CC has been used on 338 occasions. The answer response rate is 67%. In the same time span, 16 referrals have been made to the VPHC. Referrals have originated from 4/6 pilot wards. The study is ongoing so updated results will be presented.

**Conclusion**

This initiative is one of the first service evaluation protocols of its kind to be reported in the UK at the time of the COVID-19 pandemic. These are novel service developments to support the management of physical health needs jn inpatient units and understanding the implementation challenges are key to future development.

**Trial Registration**

NA

**Consent to publish**

NA

**References**

If applicable, please see guidelines

### O23 Analyzing Rwanda’s COVID-19 response using implementation science

#### Agnes Binagwaho^1^, Felix Sayinzoga^2^, Alemayehu Amberbir^1^, Kedest Mathewos^1^, Amy VanderZanden^1^, Thomas Ntawukurirayo^1^, Lisa R. Hirschhorn^1,3^

##### ^1^University of Global Health Equity, Kigali, Rwanda; ^2^Rwanda Biomedical Center, Kigali, Rwanda; ^3^Feinberg School of Medicine, Northwestern University, Chicago, IL, USA

###### **Correspondence:** Agnes Binagwaho (abinagwaho@ughe.org)

**Background**

Even though the evidence-based interventions (EBIs) for COVID-19 response were known, the success of their implementation varied globally. Rwanda was one of the successful countries, with only 26,141 cases and 344 deaths (May 16, 2021).

**Method**

We used a mixed method IS approach, with literature review, to identify strategies and contextual factors contributing to Rwanda’s successful COVID-19 response.

**Results**

More than 2 months before the first COVID-19 case, Rwanda used emerging global scientific knowledge concerning the new coronavirus and planned the equitable implementation of non-medical and medical EBIs across the country using context-specific strategies. These included leveraging existing strategies used to manage the health sector as well as facilitating contextual factors such as: the strong facility and community primary health care (PHC) system, strong community engagement, data audit and feedback, focus on enforcement of public health measures, its strong leadership, the culture of accountability, and the equity agenda. This response was accompanied by an in-depth analysis on how to mitigate the negative impacts of COVID-19 for different communities to ensure adherence to COVID-19 guidelines. Strategies included free testing, full hotel accommodation for quarantine and isolation, food and financial support to the poor, and legally delayed payments such as taxes, loans, and rent. Rwanda also used campaigns to reduce fear of seeking ordinary health services. New strategies to address access to care include preventing suspected COVID-19 cases from crossing paths with other patients, and implementing systematic testing and contact tracing across its land borders and in airports starting from the first case.

**Conclusion**

IS can help governments to put evidence into policy and practice and build a trusted, equitable strong PHC system to ensure resiliency for pandemic preparedness. IS also helps decision-makers adapt existing strategies and identify new ones to implement known EBIs according to contextual factors to successfully prepare and respond to pandemics.

### O24 Implementation of a theory-guided nursing discharge teaching intervention for adult inpatients aged 50 and over with multimorbidity: A pragmatic feasibility study

#### Joanie Pellet^1^, Marianne Weiss^2^, Cedric Mabire^1^

##### ^1^Institute of Higher Education and Research in Healthcare (IUFRS), Lausanne University Hospital and University of Lausanne, Lausanne, Switzerland; ^2^Marquette University College of Nursing, Milwaukee, Wisconsin, USA

###### **Correspondence:** Joanie Pellet (joanie.pellet@unil.ch)

**Background**

Discharge teaching should be a core nursing intervention within the overall discharge preparation [1]. Nevertheless, its implementation remains unsatisfactory in Switzerland [2]. Overcoming implementation barriers requires understanding of the nature of nurses’ behaviour to be changed and identifying implementation strategies that could effectively support these changes [3]. The objective of this study is to test the feasibility for nurses of implementing a novel discharge teaching intervention in their practice.

**Method**

This feasibility study was conducted in medical units in three hospitals in Switzerland. A sample of 13 unit nurses was recruited to be trained in and deliver the intervention. Pre-implementation, they participated in qualitative and quantitative evaluations of their teaching behaviors through focus groups and with the Determinants of Implementation Behaviour Questionnaire (DIBQ)[4]. The plan for implementation was based on the Theoretical Domains Framework and the Behavior Change Wheel of the COM-B model [5, 6].

**Results**

Mean age of nurses was 29.8 with an average of 5.1 years of work experience. Results of the DIBQ showed that socio-political context was the main barrier to discharge teaching delivery. More specifically, nurses reported a lack of support and resources from the organization. Nurses also reported having little control over teaching delivery and difficulties with planning teaching when patients are not motivated or when there is little time. These results are corroborated by the content of the three focus groups conducted with nurses. Environmental context and resources was the most reported domain influencing their behaviour regarding discharge teaching.

**Conclusion**

Results of the pre-implementation phase of this feasibility study generated an understanding of barriers and facilitators to discharge teaching delivery at the individual nurses’ level. These results provide crucial information on which behavioral determinants should be addressed by targeted implementation strategies to support the intervention implementation.

**Trial Registration**

NCT04253665

**Consent to publish**

Not applicable

**References**

1. Weiss ME, Bobay KL, Bahr SJ, Costa L, Hughes RG, Holland DE. A Model for Hospital Discharge Preparation: From Case Management to Care Transition. J Nurs Adm. 2015;45(12):606-14.

2. Mabire C, Bula C, Morin D, Goulet C. Nursing discharge planning for older medical inpatients in Switzerland: A cross-sectional study. Geriatr Nurs. 2015;36(6).

3. Michie S, van Stralen MM, West R. The behaviour change wheel: a new method for characterising and designing behaviour change interventions. Implement Sci. 2011;6:42.

4. Huijg JM, Gebhardt WA, Dusseldorp E, Verheijden MW, van der Zouwe N, Middelkoop BJ, et al. Measuring determinants of implementation behavior: psychometric properties of a questionnaire based on the theoretical domains framework. Implementation Science. 2014;9(1):33.

5. Cane J, O’Connor D, Michie S. Validation of the theoretical domains framework for use in behaviour change and implementation research. Implementation Science. 2012;7(1):37.

6. Michie S, Atkins L, West R. The behaviour change wheel : a guide to designing interventions. 2014

### P25 Implementation of Health-Justice Partnerships: A Comparative Case Study of service models in England

#### Sarah Beardon^1^, Charlotte Woodhead^2^, Silvie Cooper^1^, Hazel Genn^3^, Rosalind Raine^1^

##### ^1^Department for Applied Health Research, University College London, London, WC1E 6BT, UK; ^2^Department of Psychological Medicine, King’s College London, London, SE5 8AF, UK; ^3^Faculty of Laws, University College London, London, WC1H 0EG, UK

###### **Correspondence:** Sarah Beardon (sarah.beardon@ucl.ac.uk)

**Background**

Social welfare legal problems are harmful to physical and mental health and are increasing sharply as a result of Covid-19. Legal services can tackle health-harming social and economic conditions among patients. Partnerships between healthcare and legal services exist nationwide but implementation success is variable.

**Method**

A comparative case study was undertaken to explore service models, collaborative working and sustainability. Data were collected through semi-structured interviews (n=38) and routine service records. Qualitative analysis followed the process tracing method, with systematic cross-case comparison of themes. The analysis was informed by the General Theory of Implementation [1].

**Results**

Nine diverse health-justice partnerships across England participated, based in primary, secondary and tertiary care. Legal services were mostly co-located and delivered face-to-face but included telephone-based systems.

The extent of collaborative working between health and legal professionals was highly variable. Engagement in joint activities was influenced by: i) Willingness (positive sentiments regarding value and alignment with purpose); ii) Confidence (levels of trust, quality of relationships and formation of habits/norms); and iii) Ability (levels of knowledge, opportunities to interact and workability of systems).

Some partnerships were long-lived and ongoing, while others had ended, reduced in size or failed to establish. Resource availability was limited and the most common reason for discontinuation. Funding decisions were influenced by the inter-disciplinary nature of the partnerships, which affected funders’ perceived responsibility to support the services. Other influences included alignment with strategic goals, use of evaluation data, local prominence of the service and funder-provider relationships.

The partnerships improved access to legal assistance, achieved positive welfare outcomes and were supportive to mental wellbeing. Close collaborative working resulted in enhanced impacts for patients, staff and organisations.

**Conclusion**

Social welfare legal services provide a critical safety net for patients facing hardship and can support health services to address the health consequences of deprivation.

**Reference**

1. May, C. Towards a general theory of implementation. *Implementation Science*, 2013, 8 (1):18.

**Acknowledgements**

This study was carried out with funding from the National Institute for Health Research (NIHR) School for Public Health Research and NIHR Collaboration for Leadership in Applied Health Research and Care North Thames. The views expressed are those of the authors and not necessarily those of the NIHR or the Department of Health and Social Care.

### P26 A study to assess the scalability of an integrated falls prevention service for community-dwelling older people

#### Susan Calnan, Sheena McHugh

##### School of Public Health, University College Cork, Western Gateway Building, Western Rd, Cork, Ireland

###### **Correspondence:** Susan Calnan (susan.calnan@ucc.ie)

**Background**

The scaling up of interventions delivered in healthcare settings is a growing area in implementation research. Increasingly, the need for a phased approach is acknowledged[1],beginning with an assessment of ‘scalability’, defined as the capacity of an individual intervention to be scaled up. This study aims to assess the scalability of an integrated falls prevention service across primary and secondary care in southwest Ireland and to examine the applicability of the Intervention Scalability Assessment Tool (ISAT)[2].

**Method**

A variety of methods was used sequentially, in line with the ISAT guidance: a review of existing service data on implementation and of falls-related literature and policy documents; one-to-one interviews with key stakeholders (n=11) involved in managing the service; and an online questionnaire with stakeholders to rate scalability and provide further feedback.

**Results**

Most participants believed that the issue of falls among older people was of sufficient priority to warrant scale up of the service and that the service aligned with the health policy context in terms of prioritising falls prevention. However, considerable barriers to scale up were cited, including insufficient resources, particularly personnel, and the need for an integrated electronic patient management system linking primary and secondary care.

**Conclusion**

Notwithstanding senior management support for scaling up this service, the current scalability is questionable given the barriers that need to be addressed. Improved resourcing and ensuring that the service is more fully embedded into primary care are among the recommendations to enable future scale up of this falls prevention service to other regions in the country. The ISAT provides a systematic and structured framework for examining scalability in this context, although the detailed and technical nature of its questions require considerable time and knowledge of the service in order to complete.

**References**

1. Charif AB, Zomahoun HTV, LeBlanc A, Langlois L, Wolfenden L, Yoong SL, et al. Effective strategies for scaling up evidence-based practices in primary care: A systematic review. *Implementation Science*. 2017; 12:139

2. Milat AJ, Lee K, Conte K. et al. Intervention Scalability Assessment Tool: A decision support tool for health policy makers and implementers. *Health Research Policy and Systems*. 2020; 18:1.

### P27 Enhancing precision in the investigation of context: Study findings from Triple C (Case study research to understand Context in Complex health interventions)

#### Jamie Murdoch^1^, Sara Paparini^2^, Chrysanthi Papoutsi^2^, Sara E Shaw^2^

##### ^1^School of Health Sciences, University of East Anglia, NR4 7TJ, Norwich, UK; ^2^Nuffield Department of Primary Care Health Sciences, University of Oxford, Oxford, OX2 6GG, UK

###### **Correspondence:** Jamie Murdoch (jamie.murdoch@uea.ac.uk)

**Background**

The need for methods that successfully capture dynamic interactions between context and implementation of complex healthcare interventions is increasingly recognised. Case study (CS) approaches have the potential to provide such understanding, conducting in-depth investigations of the particularities of phenomena ‘within context’. However, what is meant by context and how it is investigated often lacks precision, potentially limiting the explanatory power of the findings beyond the specific case under investigation.

**Method**

The TRIPLE C study is funded by the Medical Research Council to develop guidance on the conduct and reporting of CS research into the influence of context on complex health interventions. Study methods include a meta-narrative literature review, a Delphi expert panel, and interviews with CS researchers. For this presentation, we will focus on findings from the meta-narrative review on the conceptualisation and operationalisation of context in empirical CS health research on complex interventions in health systems and public health.

**Results**

CS research on complex health interventions encompasses multiple perspectives grounded in different ways of viewing the world, leading to different combinations of CS designs and methods. At the time of writing we identified five meta-narratives: 1) testing complex interventions; 2) case study of organisational change; 3) realist case study evaluation; 4) naturalistic case study; and 5) case description. Conceptualisations of context ranged from the backdrop to, or factors impacting on the intervention, circumstances triggering intervention mechanisms, and socially structured practices. Overall, papers drew on a narrow selection of case study methodologists. There was limited focus on the nature and boundaries of ‘the case’ and ‘context’ and the implications of such conceptualisations for methods and knowledge production.

**Conclusion**

Deeper engagement with case study as a methodology across disciplines would enable more emphasis on aspects of context that have so far been neglected in CS research on complex health interventions.

**Trial Registration**

Non applicable

**Consent to publish**

Yes

**Reference**

Non applicable

### P28 Advancing the research infrastructure for implementation science in German speaking countries

#### Marie-Therese Schultes^1,2^, Monika Finsterwald^2^, Thekla Brunkert^3,4^, Christina Kien^5^, Lisa Pfadenhauer^6,7^, Bianca Albers^1^

##### ^1^Insititute for Implementation Science in Health Care, University of Zurich, Zurich, 8006, Switzerland; ^2^Department of Developmental and Educational Psychology, University of Vienna, Vienna, 1010, Austria; ^3^Institute of Nursing Science, Department of Public Health, University of Basel, Basel, 4056, Switzerland; ^4^University Department of Geriatric Medicine FELIX PLATTER, Basel, 4055, Switzerland; ^5^Department for Evidence-based Medicine and Evaluation, Danube University Krems, Krems, 3500, Austria; ^6^Institute for Medical Information Processing, Biometry and Epidemiology, LMU Munich, Munich, 81377, Germany; ^7^Pettenkofer School of Public Health, Munich, 81377, Germany

###### **Correspondence:** Marie-Therese Schultes (marie-therese.schultes@uzh.ch)

**Background**

To date, research activities, funding opportunities and formal education in implementation science remain concentrated on certain geographic regions, while German speaking countries (GSC) lag behind in both using and advancing implementation science. Engagement in implementation science is influenced by multiple factors, which can be inherent to individual researchers or the available research infrastructure [1]. Against this background, the *Promote ImpSci* interview study aimed at identifying challenges and facilitators for conducting implementation science in GSC.

**Method**

We conducted semi-structured interviews with nine well-established implementation researchers working in Austria, Germany and Switzerland. The interviews were held via Zoom, transcribed and analysed in MAXQDA using thematic analysis [2]. Interview topics included the interviewees’ personal experiences with challenges and facilitators for engaging in implementation science as well as their ideas for building a supportive research infrastructure.

**Results**

Challenges that became apparent in the interviews involved characteristics of implementation research projects, such as duration and costs, as well as most national funding agencies’ prioritisation of basic research. Factors that had facilitated the interviewees’ implementation research often related to their network, such as supportive mentors and international research partnerships, which were repeatedly named as a necessity to work in the field. In terms of improving the research infrastructure, the participants proposed strengthening advocacy for implementation science in academia and an increase in formal education opportunities.

**Conclusion**

Our results suggest that scientists conducting implementation research in GSC need to build a supportive network and proactively identify rare opportunities for further qualification and research funding. Building a better research infrastructure requires substantial advocacy and spokesmanship in order to convince academic decision makers of the field’s relevance. Hence, to strengthen implementation research capacity, implementation scientists - in GSC and internationally - need to join forces in advancing their discipline.

**Trial Registration**

Non applicable

**Consent to publish**

Non applicable

**References**

1. Stevens ER, Shelley D, Boden-Albala B. Perceptions of barriers and facilitators to engaging in implementation science: a qualitative study. Public Health. 2020; 185: 318-323.

2. Braun V, Clarke V. Using thematic analysis in psychology. Qual Res Psychol. 2006; 3: 77-101.

### P29 Emergency department management practices during the first year of COVID-19: A Rapid Review

#### Mickaela J Nixon^1^, Alan P Chetwynd^1^, Mona J Koshkouei^1,2^

##### ^1^Medical Sciences Division, University of Oxford, Oxford, Oxfordshire, OX3 9DU, UK; ^2^Nuffield Department of Primary Care Health Science, University of Oxford, Oxford, Oxfordshire, OX2 6HT, UK

###### **Correspondence:** Mickaela J Nixon (mickaela.nixon@medschool.ox.ac.uk)

**Background**

The coronavirus-19 pandemic (COVID-19) introduced new challenges and a need for continued adaptability in Emergency Departments (EDs) at a scale previously unseen in developed healthcare systems. Initial changes to ED management practices reactively evolved, driven by factors such as fluctuating patient demand, staffing shortages, risks of intra-hospital transmission and increasing knowledge about the virus. Innovative responses have been the rule rather than the exception.

**Method**

A rapid review of the literature was conducted to identify and understand common themes amongst ED management responses to COVID-19 and drivers of successful change. A search, limited to English language articles, was undertaken on six electronic medical databases (MEDLINE, EMBASE, AMED, CINAHL, PsycINFO, PEDro) spanning January 2020 to December 2020. Grey literature was identified using OpenGrey and PeerJ. Publications were assessed against inclusion and exclusion criteria. Relevant publications were appraised using the Critical Appraisal Skills Programme guidelines and thematically scored. Further analysis was conducted to assess prominent themes in more detail.

**Results**

The search yielded 1,938 articles. 604 papers were duplicates and 1,240 papers were deemed out of scope based on title and abstract screening. 94 papers remained, the vast majority featuring EDs in the United Kingdom and United States of America. Prominent themes of change implemented included: capacity and space management, pathways and workflow management (including triage and discharge), staffing and resourcing, communication and governance, ED-specific infection control measures, and digital innovation/ telemedicine.

**Conclusion**

This rapid review provides insight into the ED management practices implemented throughout the first year of COVID-19. The aim has been to better inform targeted, timely innovation and establish successful implementation models in response to the ongoing pandemic, as well as to provide insight for policy and practice development for large-scale public health emergencies. Findings from this review will inform the next stages of our planned fieldwork on this topic.

### P30 Preliminary results of the translation and evaluation of the Implementation Science Research Development (ImpRes) tool and supplementary guide to improving the quality of implementation research in the Brazilian context: ImpRes-BR

#### Carlos Alberto dos Santos Treichel^1^, Leidy Janeth Erazo Chavez^1^, Louise Hull^2^, Rosana Teresa Onocko Campos^1^

##### ^1^Department of Collective Health, State University of Campinas, Campinas-SP, BR; ^2^Centre for Implementation Science, Health Service and Population Research Department, Institute of Psychiatry, Psychology and Neuroscience, King’s College London, London, UK

###### **Correspondence:** Carlos Alberto dos Santos Treichel (treichelcarlos@gmail.com)

**Background**

Although Implementation Science (IS) is a field on the rise worldwide, in Brazil, it is still an incipient field. Part of this is due to the lack of accessible resources to guide the design of high-quality implementation research. Thus, researchers without expertise in IS have the difficult and time-consuming task of identifying and assimilating recommendations from different international sources to design their research. To overcome this barrier, we proposed the translation and validation of the ImpRes tool and supplementary guide [1] to improve the conceptual and methodological quality of implementation research in the Brazilian context. In this study, we present preliminary results obtained in the pilot test of the tool.

**Method**

We adhered to the compilation of good practices for cross-cultural validation of instruments and scales proposed by Sousa et al. (2010) [2]. For the pilot test, after translation, back-translation and two synthesis workshops, the tool was presented to 20 healthcare professionals and applied health researchers interested in IS, whose native language was Brazilian Portuguese. The participants were asked to review the ImpRes and rate whether each of the 10 dimensions of ImpRes-BR as clear or unclear, in addition to indicating which aspects hindered understanding.

**Results**

Two of the 10 ImpRes-BR domains were identified as unclear by more than 20% of the participants. They were: (II) Implementation Theories, Frameworks and Models (35%) and (VI) Implementation Outcomes (25%). In dimension (II), the main difficulties were related to the understanding of the concept of framework and its difference in relation to the models. In dimension (VI), the difficulties were related to the concept of adoption and sustainability.

**Conclusion**

ImpRes-BR domains that are more frequently present in other research modalities were better understood by the participants, whereas those strictly related to IS demand greater clarifications so that their content can be better assimilated by Brazilian researchers and professionals.

**Acknowledgments**

This work was supported by the São Paulo Research Foundation (FAPESP) through the grant nº 2020/14309-7.

**Trial Registration**

Non applicable

**Consent to publish**

Non applicable

**References**

1. Hull L, Goulding L, Khadjesar Z, Davis R, Healey A, Bakolis I, Sevdalis N. Designing high-quality implementation research: development, application, feasibility and preliminary evaluation of the implementation science research development (ImpRes) tool and guide. Implementation Sci. 2019;14:80.

2. Sousa VD, Rojjanasrirat W. Translation, adaptation and validation of instruments or scales for use in cross‐cultural health care research: a clear and user‐friendly guideline. J Eval Clin Pract. 2011;17(2):268-74.

### P31 A Pilot High Intensity Interval Training Intervention in inpatient mental health settings

#### Rebecca Martland^1^, Juliana Onwumere^2,3^, Brendon Stubbs^3,4^, Fiona Gaughran^1,3^

##### ^1^Department of Psychosis Studies, Institute of Psychiatry, Psychology and Neuroscience (IoPPN), King's College London, United Kingdom; ^2^Department of Psychology, Institute of Psychiatry, Psychology and Neuroscience (IoPPN), King's College London, United Kingdom; ^3^South London and Maudsley NHS Foundation Trust, London, United Kingdom; ^4^Department of Psychological Medicine, Institute of Psychiatry, Psychology and Neuroscience (IoPPN), King's College London, United Kingdom

###### **Correspondence:** Rebecca Martland (rebecca.martland@kcl.ac.uk)

**Background**

Severe mental illnesses (SMI) are associated with physical health comorbidities. Structured exercise can improve cardiometabolic health and ameliorate mental health symptomology. Focus groups with inpatients with SMI, healthcare professionals and informal carers have found positive attitudes towards high intensity interval training (HIIT) (1). A feasibility study for a HIIT intervention amongst inpatients with SMI is being conducted, to improve their physical and mental health.

**Method**

The feasibility study follows a two-part design owing to Covid-19 related adaptations to project design: a) A non-blinded randomized controlled trial (RCT) of 12 weeks of bicycle-based HIIT, delivered twice weekly, compared to treatment as usual (TAU) (Study flow diagram, Figure 1). b) A naturalistic study of inpatient HIIT; eligible participants will be invited to two sessions of HIIT per week. We will measure feasibility and acceptability of the HIIT intervention as primary outcomes, alongside secondary outcomes evaluating the physical and mental effects of HIIT. The study aims to recruit 40 patients to the RCT and 6-8 patients to the naturalistic design.

**Results**

The study is ongoing.

**Conclusion**

Exercise is a modifiable lifestyle factor that can improve cardiometabolic risk. If the HIIT intervention is found to be feasible and acceptable in psychiatric inpatient settings where embedding new ways of working can often be associated with logistic challenges, there would be scope for large scale work to evaluate the clinical, cost and implementation effectiveness of HIIT in inpatient mental health settings.

Trial registration: ClinicalTrials.gov, registration no: NCT03959735

Consent to publish

non-applicable

Reference

1. Martland R, Gaughran F, Stubbs B, Onwumere J. Perspectives on implementing HIIT interventions for service users in inpatient mental health settings: A qualitative study investigating patient, carer and staff attitudes. J Affect Disord. 2021

**Fig. 1 (abstract P31).**
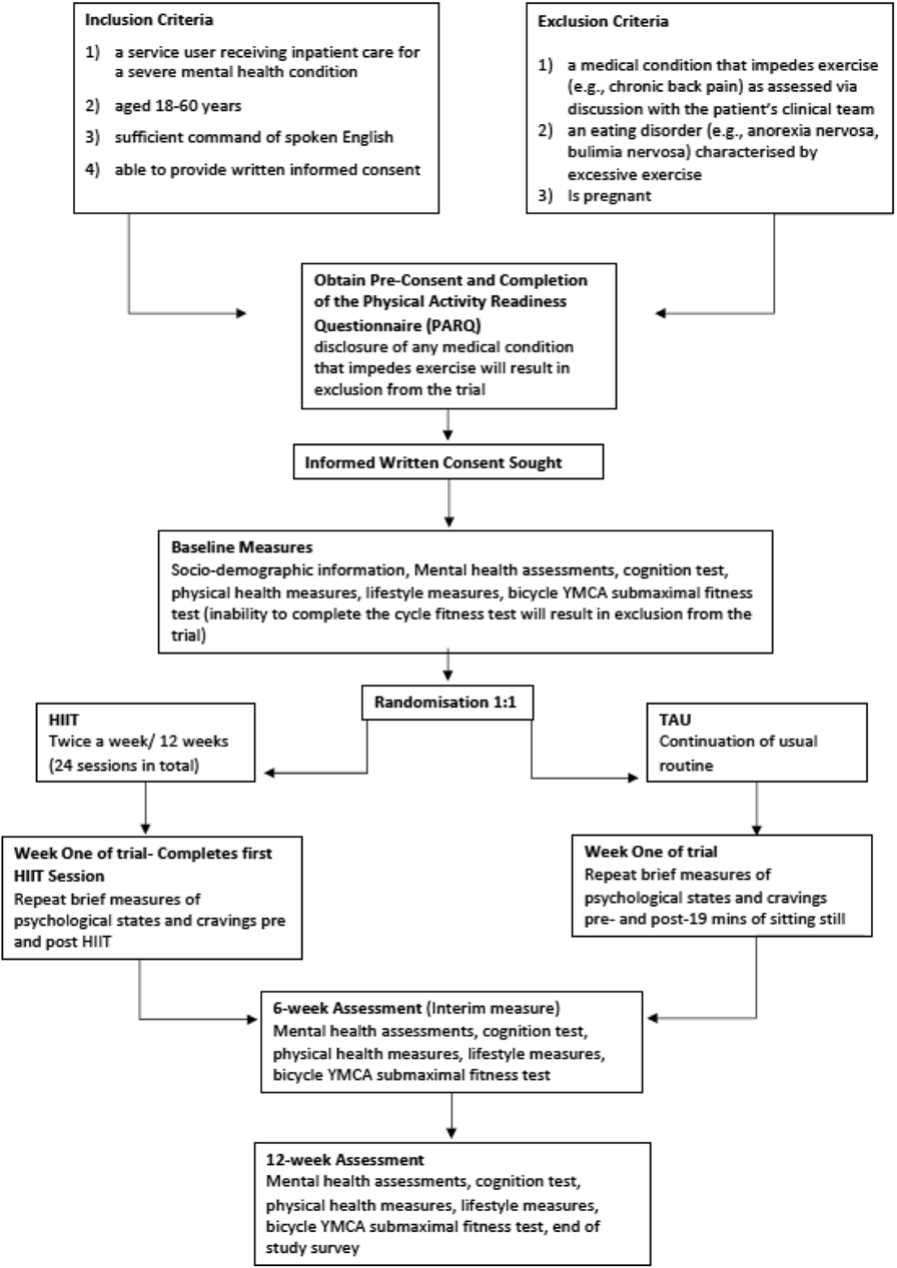
Study Flow Diagram Part A. Key words: HIIT= high intensity interval training; TAU= treatment as usual

### P33 Nursing groups’ interpretation activities in hospital wards when implementing evidence-based practice institutionalized by the hospital: A qualitative study using Organizational Learning Model

#### Keiko Ishii, Yukie Takemura, Naoko Ichikawa

##### Department of Nursing Administration, The University of Tokyo, Bunkyo, Tokyo, Japan

###### **Correspondence:** Keiko Ishii (keikoishii@g.ecc.u-tokyo.ac.jp)

**Background**

Since studies regarding the sustainability of evidence-based practice (EBP) are deficient, EBPs may not become a daily practice. We assumed the implementation of EBP in hospitals as organizational learning and in this process, which aimed to implement institutionalized rules in the clinical settings, we focused on group activities for EBP [1]. This study aimed to elucidate the nursing groups’ interpretation activities of a ward to continuing EBP institutionalized by the hospital.

**Method**

This was a qualitative study. Hospitals with 100 beds or more in the Kanto region of Japan were targeted. EBPs introduced by hospitals between 2018-2020 involving care or work by nurses were selected. Wards practicing EBPs, with five or more nurses, were studied. For data collection, semi-structured interviews with nurses and nursing leaders and participant observations were conducted, followed by qualitative content analysis.

**Results**

Interviews were conducted with 13 nurses in four wards in two hospitals. Target EBPs were pain evaluation using a pain evaluation scale, appropriate use of restrains, and excretion care using wash cream. The period from the start of EBPs in wards to the interview was three months. Participants comprised six nurses, two chief nurses, four nursing managers, and one specialist nurse. Consequently, 12 categories and 94 subcategories were generated as nursing groups’ interpretation activities in implementing EBP including ‘Efforts for staff to have the same information and knowledge about EBP’, ‘Efforts to enable staff to implement EBP in the same way’, ‘Efforts to share changes due to EBP implementation throughout the ward’ and ‘Efforts to adapt EBP to the ward’.

**Conclusion**

This study is the first to elucidate the nursing group’s interpretation activities when implementing EBPs. The results of this study could enable us to quantify interpretation activities and clarify ward-level activities that are effective in EBP sustainment.

**Consent to publish**

Informed consent from all participants to anonymously record and analyse the data was obtained before conducting the interviews. All participants consented for publication.

**Reference**

1. Crossan, M. M., Kane, H. W., White, R. E. An organizational learning framework: from intuition to institution. Academy of Management Review. 1999; 24(3):522-537.

### P34 Implementation and dissemination of home and community-based interventions for informal caregivers of people living with dementia: A systematic scoping review protocol

#### Eden M Zhu^1^, Martina Buljac-Samardžić^1^, Kees Ahaus^1^, Nick Sevdalis^2^, Robbert Huijsman^1^

##### ^1^School of Health Policy and Management, Erasmus University Rotterdam, Rotterdam, South Holland, 3062 PA, The Netherlands; ^2^Centre for Implementation Science, King’s College London, London, United Kingdom

###### **Correspondence:** Eden M Zhu (Zhu@eshpm.eur.nl)

**Background**

Ageing in place, supported by formal home and community services and informal caregivers, is the most utilized long-term care option for people with dementia. However, informal caregivers are often overwhelmed with the responsibilities of their role and could consequently suffer from negative consequences. Although many evidence-based interventions are available to support informal caregivers’ efficacy and well-being, there is a paucity of information regarding the implementation of such interventions. This scoping review aims to identify the implementation strategies, implementation outcomes, and barriers and facilitators that impede or support the uptake of interventions that support informal caregivers of people with dementia living at home.

**Method**

The search strategy has been conducted in the search engines Embase, Medline (Ovid), Web of Science, and Cochrane Central Register of Controlled trials (Wiley), followed by a three-stage screening approach. First, title and abstracts were screened by two independent reviewers using ASReview, an article screening tool using artificial intelligence. Second, full-text articles were screened by two independent reviewers and, in case of disagreement, by a third reviewer. Reference lists of the final included studies have also been checked for relevant articles. Data from the final included studies were extracted and synthesized using the Expert Recommendations for Implementing Change (ERIC) [1] compilation and Proctor’s implementation outcomes taxonomy [2] to ensure homogenous and standardized reporting of implementation information.

**Results**

Emerging results from the review will be summarized and shared at the time of the conference.

**Conclusion**

The scoping review findings will inform researchers, health service planners and practice professionals with an overview of existing literature to guide them in the effective implementation of caregiver-focused, evidenced interventions in dementia support.

**Trial Registration**

Non applicable

**Consent to publish**

Non applicable

**References**

1 Powell BJ, Waltz TJ, Chinman MJ, et al. A refined compilation of implementation strategies: results from the Expert Recommendations for Implementing Change (ERIC) project, Implement Sci 2015;10:21 doi:10.1186/s13012-015-0209-1 [published Online First: Feb 12,].

2 Proctor E, Proctor E, Silmere H, et al. Outcomes for Implementation Research: Conceptual Distinctions, Measurement Challenges, and Research Agenda, Adm Policy Ment Health 2011;38:65-76 doi:10.1007/s10488-010-0319-7 [published Online First: Mar].

### P35 The interaction of contextual factors and implementation strategies in the reduction of under-five mortality

#### Kedest Mathewos^1^, Alemayehu Amberbir^1^, Amy VanderZanden^1^, Thomas Ntawukurirayo^1^, Felix Sayinzoga^2^, Raj Kumar Subedi^3^, Fauzia Akhter Huda^4^, Lisa R Hirschhorn^1,5^, Agnes Binagwaho^1^

##### ^1^University of Global Health Equity, Kigali, Rwanda; ^2^Rwanda Biomedical Center, Kigali, Rwanda; ^3^Nepal Public Health Foundation, Kathmandu, Nepal; ^4^International Center for Diarrheal Disease Research, Bangladesh (icddr,b), Dhaka, Bangladesh; ^5^Feinberg School of Medicine, Northwestern University, Chicago, IL, USA

###### **Correspondence:** Kedest Mathewos (kmathewos@ughe.org)

**Background**

Countries have achieved variable success in reducing under-five mortality (U5M) [1]. Evidence-based interventions (EBIs) to reduce amenable U5M are known and relevant to most settings. However, countries’ ability to successfully implement them relies on their understanding of national and subnational context and use of context-informed implementation strategies.

**Method**

We used a mixed methods implementation science approach driven by a hybrid framework and theory of change to identify how six Exemplar countries that outperformed their regional and economic peers in the reduction of U5M – Bangladesh, Ethiopia, Nepal, Peru, Rwanda, Senegal – were able to implement EBIs to reduce U5M between 2000 and 2015 [2].

**Results**

Contextual factors at global, national, MOH/ health systems, and community/individual levels affected the choice, design, and execution of the implementation strategies (Table 1). Factors including national priority for U5M reduction and strong culture of data use facilitated a multisectoral response and data use for decision-making respectively. Countries faced with challenging contextual factors adapted their strategies or adopted new ones to address barriers. For instance, when geographic barriers presented a challenge to equitable healthcare delivery in Bangladesh, Ethiopia, Nepal, and Senegal, these countries adopted community-based delivery of health services and built new facilities. Using context-informed implementation strategies, these countries achieved various targeted implementation outcomes such as acceptability, reach, and sustainability, and ultimately contributed to U5M reduction.

**Conclusion**

Efforts to understand their context and consequently choose and adapt appropriate implementation strategies helped the Exemplar countries to achieve notable progress in implementing EBIs contributing to reducing U5M between 2000 and 2015.

**References**

1. Suzuki E. In 2015, the global child mortality rate is less than half its 1990 levels, but the MDG 4 target has not been met [Internet]. World Bank Blogs. 2015 [cited 2021 May 12]. Available from: https://blogs.worldbank.org/opendata/2015-global-child-mortality-rate-less-half-its990-levels-mdg-4-target-has-not-been-met

2. Hirschhorn LR, Frisch M, Ntawukuriryayo JT, VanderZanden A, Donahoe K, Mathewos K, et al. Development and application of a hybrid implementation research framework to understand success in reducing under-5 mortality in Rwanda. Gates Open Res [Internet]. 2021 Mar 29 [cited 2021 May 12];5:72. Available from: 10.12688/gatesopenres.13214.1

**Table 1 (abstract P35).** Interaction between selected contextual factors and implementation strategies

Level	Contextual factor	Potential impact on implementation of EBIs	Selected implementation strategies
Global	Donor funding priorities and availability (Facilitator in all countries but was a barrier at times in Senegal)	*Facilitator*: If efficient and coordinated, results in the prioritization of U5M and provides the financing. *Barrier*: Could be a barrier if donors and national priorities are not aligned and if the funding is limited or time constrained.	• Leveraging donor support • Donor coordination • Government financing
National/ subnational	Existing national priority for health including U5M (Facilitator in all countries)	*Facilitator*: Sustained commitment to health allows for accountable EBI implementation. *Barrier*: When absent, can lead to reduced accountability. Leadership in MOH is key.	Multisectoral approach National leadership and accountability for EBI and U5M
MOH/health systems	Community health system and structure (Facilitator in all)	*Facilitator:* If present and strong, it can bridge the gap between the healthcare system and communities. *Barrier*: If the community health workers are not well trained and the system is weak, it can lead to gaps in implementation and affect sustainability.	• Community engagement • Community education and sensitization • Community-based care delivery • Leveraging existing systems • Focus on equity
Community/ individual	Culture and beliefs (Barrier in Nepal, Ethiopia and Peru, facilitator in Rwanda and Senegal, both in Bangladesh)	*Facilitator*: Culture and beliefs can facilitate acceptance of EBIs. *Barrier*: If a barrier, this contextual factor can lead to low uptake of EBIs.	• Local stakeholder engagement • Community education and sensitization • Community engagement • Data use for prioritization • Focus on equity

### P36 Development of an online implementation outcome repository

#### Louise Hull^1^, Zarnie Khadjesari^2^, Nick Sevdalis^1^

##### ^1^Centre for Implementation Science, Health Service and Population Research Department, Institute of Psychiatry, Psychology and Neuroscience, King’s College London, London, UK; ^2^Behavioural and Implementation Science Research group, School of Health Sciences, University of East Anglia, Norwich, UK

###### **Correspondence:** Louise Hull (louise.hull@kcl.ac.uk)

**Background**

To support the use of precise and accurate implementation outcome (IO) instruments, we conducted a systematic review to identify and appraise studies that assess the measurement properties of quantitative IO instruments used in physical healthcare settings.[1] Following the review, our aim was to mobilise the reviewed evidence so that is it accessible to all stakeholder groups. We report the development of an online IO repository.

**Method**

We worked closely with Icon Creative Designs [2] to develop the repository. During the design phase we sought feedback from implementation stakeholders, including researchers, healthcare practitioners and patients and the public, on the content, design, and usability of the repository.

**Results**

The repository allows users to:

- Search for IO instruments, included in Proctor’s IO taxonomy [3]
- View a summary of the instrument, the number of items, the country of application and the level of analysis (e.g., patient, provider, organisation)
- Examine the methodological quality assessment of the psychometric study, based on the COSMIN checklist [4]
- View the psychometric quality assessment of each instrument, based on the ConPsy checklist [5]
- See the usability rating of the instrument and where permission is granted, access both the psychometric study and the published instrument

The repository includes 55 IO instruments. See Figure 1 for an example of an instrument summary page.

**Conclusion**

Based on a systematic review of the literature, we have created an online resource to promote widespread accessibility for implementation stakeholders wishing to quantitatively measure IOs.

**Trial Registration:**

Non applicable.

**Consent to publish**

Yes

**References**

1. Khadjesari Z, Boufkhed S, Vitoratou S, Schatte L, Ziemann A, Daskalopoulou C, Uglik-Marucha E, Sevdalis N, Hull L. Implementation outcome instruments for use in physical healthcare settings: a systematic review. Implement Sci. 2020;15(1):66.

2. Icon Creative Designs. [cited 2021 September 30] Available from: https://www.iconcreativedesign.com/.

3. Proctor E, Silmere H, Raghavan R, Hovmand P, Aarons G, Bunger A, Griffey R, Hensley M. Outcomes for implementation research: conceptual distinctions, measurement challenges, and research agenda. Admin Pol Ment Health. 2011;38(2):65–76.

4. Terwee CB, Mokkink LB, Knol DL, Ostelo RWJG, Bouter LM, de Vet HCW. Rating the methodological quality in systematic reviews of studies on measurement properties: a scoring system for the COSMIN checklist. Qual Life Res Int J Qual Life Asp Treat Care Rehab. 2012;21(4):651–7.

5. Psychometrics and Measurement Lab, King’s College London [cited 2021 September 30] Available from: Psychometrics & Measurement Lab (kcl.ac.uk).

**Fig. 1 (abstract P36).**
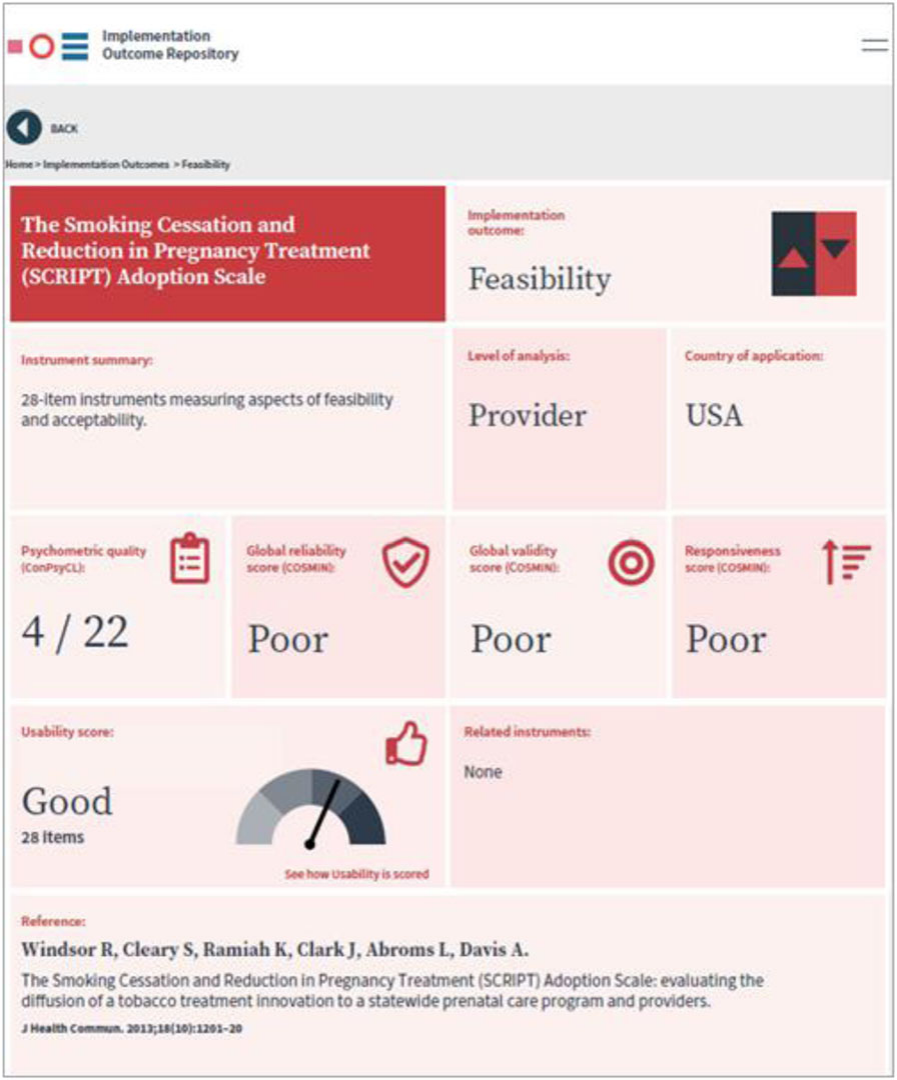
Implementation outcome repository instrument summary page example

### P37 Defining, describing and assessing pragmatic qualities of quantitative instruments measuring implementation determinants and outcomes: A scoping and critical review of the literature

#### Louise Hull^1^, Richard Boulton^2^, Fiona Jones^2^, Annette Boaz^3^, Nick Sevdalis^1^

##### ^1^Centre for Implementation Science, Health Service and Population Research Department, King’s College London, London, UK; ^2^Centre for Health and Social Care, St George's, University of London and Kingston University, London, UK; ^3^Faculty of Public Health & Policy, London School of Hygiene & Tropical Medicine, London, UK

###### **Correspondence:** Louise Hull (louise.hull@kcl.ac.uk)

**Background**

The pragmatic quality (i.e., brief, low cost, reliable, valid) of quantitative implementation measures has received increased attention in the implementation science literature. This study sought to identify and critically appraise published studies to understand (i) how pragmatism is defined as a measurement construct/quality of implementation determinants and outcome instruments; (ii) how pragmatic qualities of instruments are appraised; and (iii) identify key gaps and limitations of the current evidence-base.

**Method**

We conducted a scoping review of the literature also employing methods of critical review. We searched PubMed and PsycINFO databases, using the OVID interface, for relevant articles published between January 2010 and September 2020. The following search strategy: [Pragmatic AND Assessment* OR measure* OR instrument* OR questionnaire* OR survey* AND implementation NOT language] was employed at the title and abstract level.

**Results**

The search retrieved 731 articles. 680 articles were excluded at title and abstract stage, resulting in 52 full-text articles assessed for eligibility. A further 41 articles were excluded at full-text stage, resulting in 9 articles meeting inclusion criteria. Three of the nine articles involved international stakeholders in conceptualising and defining pragmatism. The same three articles employed specific methodologies to define pragmatism, including a systematic review of the literature, stakeholder interviews, concept mapping, and a Delphi process. One article assessed the pragmatic qualities, above and beyond the psychometric qualities, of implementation measures, using the Psychometric and Pragmatic Evidence Rating Scale (PAPERS).[1]

**Conclusion**

Although the evidence base within the implementation literature on what pragmatism is and how it might be assessed is limited, some of the work identified in the review provides a strong foundation to build upon, by testing its applicability in other settings and among a more diverse group of stakeholders. We discuss directions for further development of the concept of pragmatism.

**Trial Registration**

Non applicable

**Consent to publish**

Yes

**Reference**

1. Stanick CF, Halko HM, Nolen EA, Powell BJ, Dorsey CN, Mettert KD, Weiner BJ, Barwick M, Wolfenden L, Damschroder LJ, Lewis CC. Pragmatic measures for implementation research: development of the Psychometric and Pragmatic Evidence Rating Scale (PAPERS). Transl Behav Med. 2021;11(1):11-20.

### P38 A feasibility hybrid II randomised controlled trial of volunteer ‘Health Champions’ supporting people with serious mental illness manage their physical health

#### Julie Williams^1^, Ray McGrath^2^, Ubong Akpan^2^, Isobel Mdudu^2^, Fiona Gaughran^3^, Ioannis Bakolis^4^, Andy Healey^5^, Zarnie Khadjesari^6^, Euan Sadler^7^, Boryana Smilenova^2^, Nick Sevdalis^1^

##### ^1^Centre for Implementation Science, King’s College London, London, UK; ^2^South London and Maudsley NHS Foundation Trust, London, UK; ^3^Psychosis Studies, King's College London, London, UK; ^4^Department of Biostatistics and Health Informatics, King’s College London, London, UK; ^5^Kings Health Economics, King's College London, London, UK; ^6^Behavioural and Implementation Science (BIS) research group, University of East Anglia, Norwich, UK; ^7^Department of Nursing, Midwifery and Health, University of Southampton, Southampton, UK

###### **Correspondence:** Julie Williams (julie.williams@kcl.ac.uk)

**Background**

People with serious mental illnesses (SMI) such as schizophrenia often also have physical health illnesses and reduced life expectancy. It has been recognised as an important area to be addressed. Supporting people individually in managing their physical health is one area of research. Research has shown that volunteers are able to support people with SMI in other areas eg wellbeing. This intervention matching specially trained volunteers with people using mental health services to support them with their physical health. We describe a novel intervention called Health Champions.

**Method**

The study is a feasibility randomised Hybrid II controlled trial. The intervention involves training volunteers to be ‘Health Champions’ to support individuals with SMI using community mental health services. Health Champions provide one-to-one support weekly for up to nine months following the initial introduction. We are aiming to recruit 120 participants with half of participants having a Health Champion and half having treatment as usual. We are evaluating the implementation of the intervention with our primary outcome being the acceptability of the intervention. We are also evaluating the cost-effectiveness of the intervention. Our primary effectiveness outcome is physical health related quality of life and we are also collecting data on other related clinical and social outcome.

The intervention has been amended so that we could deliver it remotely during the COVID pandemic with the aim of it being face-to-face when this is able to happen.

**Results**

The study is underway. We will discuss the progress of the trial so far and our learning from delivering the intervention during the COVID pandemic.

**Conclusion**

Providing support to people with SMI with their physical health is even more important during the COVID pandemic. Our study will give us good data on the challenges of implementation during a global pandemic.

**Trial Registration**

ClinicalTrials.gov, registration no: NCT04124744

**Consent to publish**

NA

### P39 What are the strategies for implementing primary care models in maternity? A systematic review on midwifery units

#### Laura Batinelli^1^, Ellen Thaels^2^, Nathalie Leister^1^, Christine McCourt^1^, Manila Bonciani^3^, Lucia Rocca-Ihenacho^1^

##### ^1^Centre for Maternal and Child Health Research, School of Health Sciences, City, University of London, 1 Myddelton Street, London EC1R 1UW, United Kingdom; ^2^Faculty of Health & Wellbeing, School of Community Health and Midwifery, University of Central Lancashire, UCLAN, Brook Building, Victoria Street, Preston PR17QT United Kingdom; ^3^Management and Healthcare Laboratory, Sant’Anna School of Advanced Studies, Piazza Martiri della Libertà 33, 56127 Pisa, Italy

###### **Correspondence:** Laura Batinelli (laura.batinelli@city.ac.uk)

**Background**

Midwifery Units (MUs) are associated with optimal perinatal outcomes, improved service users’ and professionals’ satisfaction as well as being the most cost-effectiveness option. However, they still do not represent the mainstream option of maternity care in many countries [1, 2]. Understanding effective strategies to integrate this model of care into maternity services could support and inform the MU implementation process that many countries and regions still need to approach.

**Method**

A systematic search and screening of qualitative research about implementation of new MUs was conducted (Prospero protocol reference: CRD42019141443) using PRISMA guidelines [3]. Included articles were appraised using the CASP checklist [4]. A meta-synthesis approach to analysis was used [5]. No exclusion criteria for time or context were applied to ensure inclusion of different implementation attempts even under different historical and social circumstances. A sensitivity analysis was conducted to reflect the major contribution of higher quality studies.

**Results**

Twelve studies were identified for inclusion in this review after a screening process (see figure 1). The synthesis highlighted two broad categories: drivers to open the new MUs and barriers or facilitators to the MU implementation. The latter category included eight key themes: “culture and perceptions”, “healthcare system”, “midwives’ identity and role”, “knowledge, skills and training”, “leadership”, “collaborative approach”, “integration” and “environment”. A logic model was created to explain the role of each during the implementation process.

**Conclusion**

The studies selected were from a range of settings and time periods and used varying strategies. Nonetheless, consistencies were found across different implementation processes. These findings can be used in the systematic scaling up of MUs and can help addressing barriers at system, service and individual levels. All three levels need to be addressed when implementing this type of change.

**References**

1. Scarf VL, Rossiter C, Vedam S, Dahlen HG, Ellwood D, Forster D, Foureur MJ, McLachlan H, Oats J, Sibbritt D, Thornton C. Maternal and perinatal outcomes by planned place of birth among women with low-risk pregnancies in high-income countries: a systematic review and meta-analysis. Midwifery. 2018 Jul 1;62:240-55.

2. Hollowell J, Rowe R, Townend J, Knight M, Li Y, Linsell L, Redshaw M, Brocklehurst P, Macfarlane A, Marlow N, McCourt C. The Birthplace in England national prospective cohort study: further analyses to enhance policy and service delivery decision-making for planned place of birth.

3. Moher D, Shamseer L, Clarke M, Ghersi D, Liberati A, Petticrew M, Shekelle P, Stewart LA. Preferred reporting items for systematic review and meta-analysis protocols (PRISMA-P) 2015 statement. Systematic reviews. 2015 Dec;4(1):1-9.

4. Critical Appraisal Skills Programme (CASP) 2014. Available from: http://media.wix.com/ugd/dded87_29c5b002d99342f788c6ac670e49f274.pdf [Accessed 06 May 2021]

5. Thomas J, Harden A. Methods for the thematic synthesis of qualitative research in systematic reviews. BMC medical research methodology. 2008 Dec;8(1):1-0.

**Fig. 1 (abstract P39).**
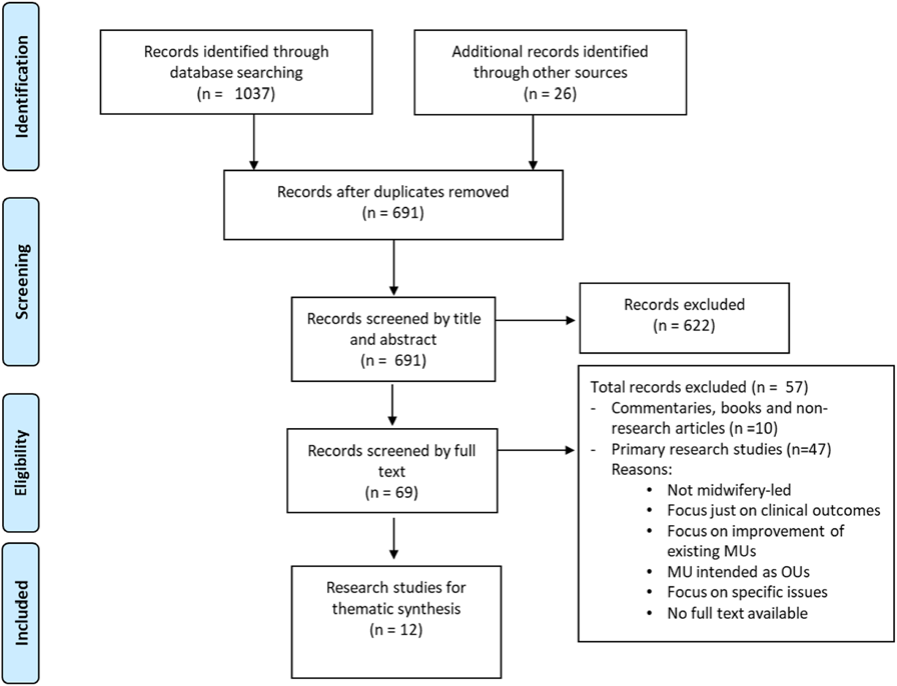
Screening process using PRISMA flowchart

### P40 Factors effecting the sustainability of NSPCC services adopted by Local Authorities and voluntary organisations

#### Hayley Clark, Emma Smith

##### NSPCC, Weston House, 42 Curtain Rd, London EC2A 3NH, UK

###### **Correspondence:** Emma Smith (emma.smith@NSPCC.org.uk)

**Background**

The NSPCC Scale Up Unit works with Local Authorities and voluntary organisations to help them successfully adopt ‘tried and tested’ NSPCC services in order that more children can potentially be helped. Much research exists on the implementation of services in the early stages, including NSPCC services [1]. However, there is little evidence, at least within the social care field, about how services are sustained in the medium to longer term [2]. Therefore, the current research aimed to find out about how NSPCC services were fairing two to four years after they received their initial training, and to identify the factors affecting the extent to which they were considered sustainable.

**Method**

Three well established NSPCC services were included in this study. Qualitative interviews were conducted with 21 professionals from each sites (seven from each service) and three interviews were carried out with the NSPCC implementation manager of each service. Sites were categorised (See Table 1) according to how sustainable they were judged to be on a range of criteria influenced by Fixsen’s (2007) Implementation principles [3]. Interviews were analysed thematically using the Framework approach.

**Results**

Factors which appeared to help sites be more sustainable included having a strong lead/champion(s), strategic buy-in and good partnership working. Sites that had built in strategies such as a train the trainer model, to protect the service against issues such as staff turnover were more likely to be successful. Factors negatively impacting on sustainability included having time limited funding and no protected time for the service.

**Conclusion**

The findings were mixed but overall positive, suggesting that many NSPCC services were well sustained over time. Recommendations of how the NSPCC could better support sites at an earlier stage are considered.

**Trial Registration**

N/A

**Consent to publish**

N/A

**References**

1. Smith E, Johnson R, Andersson T, Belton E, Kyriacou S, Hodson D. Evaluating the Graded Care Profile 2: Comparisons with the Original Tool and Factors Affecting Uptake and Use of the Updated Tool. Child Abus Rev [Internet]. 2019 Jul 1;28(4):299–309. Available from: https://doi.org/10.1002/car.2570

2. Wiggins, M., Austerberry, H. and Ward H. Implementing Evidence-Based Services in Children’s Services: Key Issues for Success. 2012.

3. Fixsen DL, Blase KA, Naoom SF, Van Dyke M, Wallace F. Implementation: The missing link between research and practice. NIRN Implement Br. 2009;1:218–27.

**Table 1 (abstract P40).** Level of sustainability

Service Name	Low	Medium	High
Graded Care Profile 2	2	1	3
Domestic Abuse Recovering Together	3	1	3
Baby Steps	1	0	5
**Total**	**6**	**2**	**11**

### P41 Protocol for developing, implementing, and evaluating an intervention designed to support the safe (re)integration of unpaid caregivers into Canadian long-term care homes during the COVID-19 pandemic

#### Natasha L Gallant^1^, Atul Jaiswal^2^, Afra Mehwish^1^, Laura Daari-Herman^1^, Ivy Cheng^3^, Chi-Ling Joanna Sinn^4^, Iwona Bielska^5^, Catherine Aubrecht^6^, Heather Finnegan^7^, Duyen Nguyen^8^, Sara Shearkhani^9^, Emily Moore^10^, Aislinn Conway^11^, Logan Lawrence^12^, El Kebir Ghandour^13^, Malcolm Doupe^7^, Thomas Hadjistavropoulos^1^, Walter Wodchis^9^, Jacqueline Gahagan^14^, Gina Agarwal^5^, Adrienne Chan^3^, Merrick Zwarenstein^15^

##### ^1^Department of Psychology, University of Regina, Regina, Saskatchewan, Canada; ^2^School of Optometry, Université de Montreal, Montreal, Quebec, Canada; ^3^Sunnybrook Health Sciences Centre, Toronto, Ontario, Canada; ^4^Faculty of Applied Health Sciences, University of Waterloo, Waterloo, Ontario, Canada; ^5^Department of Health Research Methods, Evidence, and Impact, McMaster University, Hamilton, Ontario, Canada; ^6^Department of Sociology, St. Francis Xavier University, Antigonish, Nova Scotia, Canada; ^7^Department of Community Health Sciences, University of Manitoba, Winnipeg, Manitoba, Canada; ^8^Government of New Brunswick, Fredericton, New Brunswick; ^9^Institute of Health Policy, Management and Evaluation, University of Toronto, Toronto, Ontario, Canada; ^10^Department of Psychology, McGill University, Montreal, Quebec, Canada; ^11^BORN Ontario, Ottawa, Ontario, Canada; ^12^Nova Scotia Health Association, Halifax, Nova Scotia, Canada; ^13^Département de médecine familiale et d'urgence, Université Laval, Quebec City, Quebec, Canada; ^14^School of Health and Human Performance, Dalhousie University, Halifax, Nova Scotia, Canada; ^15^Schulich School of Medicine & Dentistry, Western University, London, Ontario, Canada

###### **Correspondence:** Natasha L Gallant (Natasha.Gallant@uregina.ca)

**Background**

Supports for unpaid caregivers (e.g., family, volunteers, staff) in long-term care (LTC) homes as essential partners in care have been largely overlooked in Canada’s response to the COVID-19 pandemic [1]. Thus, a protocol for an intervention designed to support the safe (re)integration of unpaid caregivers into LTC homes during the COVID-19 pandemic has been presented. Based on policy recommendations from Healthcare Excellence Canada [2], this intervention will prepare unpaid caregivers for and facilitate their entry into LTC homes.

**Method**

With each of our three partnering LTC homes, the intervention will be co-developed with standing committees comprised of residents, unpaid caregivers, and staff. The intervention will be implemented using a rapid-cycle quality improvement strategy (i.e., Plan-Do-Study-Act [PDSA]). A non-randomized controlled before-and-after study design using a mixed-methods approach will also be used to evaluate the intervention. Furthermore, to support the development, implementation, and evaluation of our intervention, guidance documents have been created to support meaningful engagement of residents, unpaid caregivers, and staff; inform integrated and end-of-grant knowledge translation and exchange; and employ equity, diversity, and inclusion practices.

**Results**

Following the implementation of our intervention, we expect residents to report reduced levels of loneliness and greater perceived social support; unpaid caregivers to report more meaningful involvement with residents; and staff to report reduced levels of burnout and moral distress. An exploration of the study design will allow for the identification of possible opportunities for improvements and mitigation of potential challenges related to our protocol.

**Conclusion**

This protocol serves as a framework outlining an approach to developing, implementing, and evaluating an intervention supporting unpaid caregivers within the context of COVID-19. Findings from this program of research are also likely to support future work to support the presence of unpaid caregivers during future outbreaks of COVID-19 or other infections in LTC homes.

**Trial Registration**

Non applicable

**Consent to Publish**

Non applicable

**References**

1. Stall NM, Johnstone J, McGreer AJ, Dhuper M, Dunning J, Sinha SK. Finding the right balance: An evidence-informed guidance document to support the re-opening of Canadian long-term care homes to family caregivers and visitors during the COVID-19 pandemic. JAMDA. 2020;21:1365-1370.

2. Healthcare Excellence Canada. Policy Guidance for the Reintegration of Caregivers as Essential Care Partners [Internet]. [cited 2021 May 14]. Available from: https://www.cfhi-fcass.ca/innovations-tools-resources/item-detail/2020/11/23/Essential-Care-Partners-Policy-Guidance

### P42 Web App for mental health support during the COVID-19 pandemic and general lockdown: lessons learned from an urgent development and implementation

#### Sara Guila Fidel-Kinori^1,2^, Gerard Carot-Sans^3,4^, Andrés Cuartero-Barbanoj^5,3^, Damià Valero-Bover^3,4^, Jordi Piera-Jimenez^3,4,6^, Rosa Romà-Monfà^3^, Elisabet Garcia-Ribatallada^3^, Pol Perez-Sust^3,7^, Jordi Blanch-Andreu^3^, Josep Antoni Ramos-Quiroga^1,2^

##### ^1^Hospital Universitari Vall d’Hebron, Barcelona, Spain; ^2^Universitat de Autònoma de Barcelona, Bellaterra, Spain; ^3^Servei Català de la Salut, Barcelona, Spain; ^4^Digitalization for the Sustainability of the Healthcare System (DS3), Sistema de Salut de Catalunya, Barcelona, Spain; ^5^Sistema d’Emergències Mèdiques, L'Hospitalet de Llobregat, Spain; ^6^Open Evidence Research Group, Universitat Oberta de Catalunya, Barcelona, Spain; ^7^Departament de Salut, Barcelona, Spain

###### **Correspondence:** Gerard Carot-Sans (gerard.carot@gencat.cat)

**Background**

In March 2020, the Spanish government dictated a strict lockdown to contain the COVID-19 spread that lasted three months. The Catalan health department urged the development and implementation of a mobile solution to provide the general population with support for coping with the emotional struggle associated with quarantine [1,2] and facilitate reaching healthcare professionals. We found no previous evidence on mechanisms for implementing digital health solutions for mental health targeting the general population [3].

**Method**

The WebApp was developed (adapted from the PTSD Coach App [4]) and launched between March 26 and April 15, 2020. A special unit of 80 trained psychologists was incorporated into the emergency medical service to facilitate implementing contact services through the WebApp, which proactively offered professional phone support to users with severe anxiety and depression symptoms. Social networks and TV/radio advertisements were used to disseminate the WebApp among the general population. Implementation was measured by the number of accesses to the WebApp and the number of successful contacts with healthcare professionals.

**Results**

From launch to December 2020, 470,063 users accessed the WebApp, mainly during the first wave (80.9%) (Figure 1). In the first wave, press releases regarding critical events of the pandemic progression and government decisions on containment measures were followed by a utilization peak, irrespective of the sense of the information (i.e., positive or negative). The second wave was characterized by a lower and less responsive utilization of the Web App. Overall, 75,347 phone calls were offered; successful phone calls ranged from 10.8% (first wave) to 17.0% (between-waves) of all individuals to whom phone contact was offered because of severe symptoms.

**Conclusion**

The evolving and rapidly changing context of the emergency context (e.g., reactivity to news, pandemic fatigue) should be considered when implementing mobile health solutions for the general population in this scenario.

**Acknowledgements**

The authors would like to thank all the teams involved in the development of the technological solution and the delivery of the intervention from Servei Català de la Salut, Centre de Telecomunicacions i Tecnologies de la Informació, Sistema d’Emergències Mèdiques and Departament de Salut. We also thank the developers of the Post-Traumatic Stress Disorder (PTSD) Coach App for giving us permission for adapting and translating the App.

**References**

1. Xiong J, Lipsitz O, Nasri F, Lui LMW, Gill H, Phan L, et al. Impact of COVID-19 pandemic on mental health in the general population: A systematic review. J. Affect. Disord. Elsevier; 2020;277:55–64.

2. Brooks SK, Webster RK, Smith LE, Woodland L, Wessely S, Greenberg N, et al. The psychological impact of quarantine and how to reduce it: rapid review of the evidence. Lancet. Lancet Publishing Group; 2020;395:912–20.

3. Lewis CC, Boyd MR, Walsh-Bailey C, Lyon AR, Beidas R, Mittman B, et al. A systematic review of empirical studies examining mechanisms of implementation in health. Implement. Sci. Implementation Science; 2020;15:1–25.

4. Kuhn E, Greene C, Hoffman J, Nguyen T, Wald L, Schmidt J, et al. Preliminary evaluation of PTSD Coach, a smartphone app for post-traumatic stress symptoms. Mil. Med. Oxford Academic; 2014;179:12–8.

**Fig. 1 (abstract P42).**
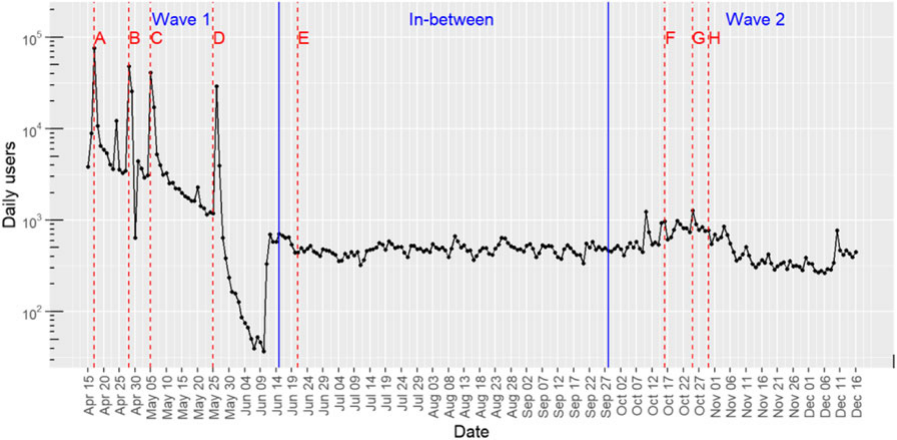
Longitudinal analysis of the number of accesses to the webapp (logarithmic scale)

### P43 Reducing the equity gap in under-5 mortality through an innovative community health program in Ethiopia: An implementation research study

#### Alemayehu Amberbir^1^, Laura Drown^2^, Alula M. Teklu^3^, Kedest Mathewos^1^, Jovial Thomas Ntawukuriryayo^1^, Agnes Binagwaho^1^, Lisa R. Hirschhorn^4^

##### ^1^University of Global Health Equity, Kigali, Rwanda; ^2^Division of Global Health Equity at Brigham and Women's Hospital in Boston, MA, USA; ^3^MERQ Consultancy PLC, Addis Ababa, Ethiopia; ^4^Feinberg School of Medicine, Northwestern University, Chicago, IL, USA

###### **Correspondence:** Alemayehu Amberbir (aamberbir@ughe.org)

**Background**

Ethiopia significantly dropped its under-5 mortality (U5M) as compared to countries in the region. One of the challenges that Ethiopia addressed to achieve was inequity through the implementation of the national community health program - the Health Extension Program (HEP). This study aims to understand how Ethiopia leveraged the HEP program to address inequities at a national level.

**Method**

This study was part of a larger six-country case study series [1] designed using implementation research to understand how countries implemented evidence-based interventions (EBIs) known to reduce U5M. We used mixed methods study informed by a hybrid implementation science framework to understand the progress (or lack thereof) of coverage of chosen EBIs.

**Results**

Ethiopia implemented most of the prevention and curative EBIs known to address leading causes of U5M, despite the challenges in achieving equity. This was particularly evident for pastoralist and rural communities. Ethiopia’s success represents a combination of the implementation and expansion of many EBIs as well as broader contextual factors including health systems strengthening, water sanitation, and hygiene, nutrition, pro-poor interventions, strong intersectoral collaboration and improvement in women’s literacy. The most common implementation strategies Ethiopia utilized include: its national policy and development planning, well-coordinated partner support, using data for decision-making and utilizing the HEP. The HEP served as a platform for delivery of the EBIs with health extension workers playing a key role. This strategy improved feasibility and scale up of EBIs and addressed challenges related to access. Inequity, however remains to be a challenge in pastoralist communities because of contextual factors related to their mobility and sparse distribution.

**Conclusion**

Leveraging the HEP as a platform for service delivery allowed Ethiopia to address inequity and successfully introduce and scale new EBIs at a national level. Additional effort is required to reduce equity gap among pastoralist communities.

**Reference**

1. Binagwaho A, Teklu AM, Drown L, Udoh K, Frisch M, Ntawukuriryayo JT, et al. Exemplars in Under-5 Mortality: Ethiopia Case Study [Internet]. 2020 [cited 2021 May 13]. Available from: https://www.exemplars.health/-/media/files/egh/resources/underfive-mortality/ethiopia/ethiopia-case-study-_-final-_10042020.pdf

### P44 Protocol for a hybrid II study exploring the feasibility of delivering, evaluating, and implementing a self-management programme for people with neuromuscular diseases at a specialist neuromuscular centre (ADAPT-NMD)

#### Laurence E Lee^1^, Stefan T Kulnik^2^, Annette Boaz^3^, Geoffrey M Curran^4^, Gita M Ramdharry^1^

##### ^1^Department of Neuromuscular Diseases, University College London, London, UK; ^2^Faculty of Health Social Care and Education, Kingston University and St George’s University of London, London, UK; ^3^Faculty of Public Health and Policy, The London School of Hygiene & Tropical Medicine, University of London, London, UK; ^4^Departments of Pharmacy Practice and Psychiatry, University of Arkansas for Medical Sciences, Arkansas, USA

###### **Correspondence:** Laurence E Lee (louie.lee@ucl.ac.uk)

**Background**

Self-management support (SMS) forms a central pillar in the management of many long-term conditions. It is firmly aligned with national health policy ^[1,2^], but has little exploration in neuromuscular diseases (NMDs). Bridges is a SMS programme originally developed in stroke ^[3]^. A new version for NMDs (Neuromuscular Bridges) has recently been co-designed but requires evaluation. The implementation of SMS is inherently complex with potential barriers at the level of the patient, provider, and wider organisation. The success of implementing programmes can be highly dependent on context, indicating a rationale for considering implementation determinants at an early stage^[ 2]^. This study aims to explore the feasibility of delivering and evaluating Neuromuscular Bridges, whilst simultaneously testing the feasibility of an implementation strategy bundle for a specialist neuromuscular centre.

**Method**

This study employs a hybrid II[4] design underpinned by Normalisation Process Theory ^[5]^ (NPT), which has been used prospectively to inform the implementation plan and will be invoked in the analysis. The feasibility of delivering and evaluating Neuromuscular Bridges will be assessed using a single-arm pre-post design. We will explore acceptability, demand within the service, performance of outcome measures, recruitment and retention. Implementation strategies have been selected from a refined taxonomy of strategies ^[6]^, mapped to NPT, and targeted at known barriers and facilitators at the specialist centre that were identified from preliminary stakeholder engagement activities. The impact of the strategy bundle on fidelity, acceptability, appropriateness and adoption will be evaluated using qualitative interviews, administrative data, surveys, and a notes audit.

**Results**

This is a protocol, so no results are available yet.

**Conclusion**

This this study hopes to provide valuable feasibility data on a co-designed SMS programme for people with NMDs and enhance understandings of factors and requirements for delivering, evaluating, and implementing it at a specialist centre.

**References**

1. National Health Service (NHS). NHS Long term plan [Internet]. NHS England. 2019 [cited 2020 Sep 27]. Available from: https://www.longtermplan.nhs.uk/

2. Dineen-Griffin S, Garcia-Cardenas V, Williams K, Benrimoj SI. Helping patients help themselves: A systematic review of self-management support strategies in primary health care practice. PLoS One. 2019;14(8).

3. Jones F, Mandy A, Partridge C. Changing self-efficacy in individuals following a first time stroke: Preliminary study of a novel self-management intervention. Clin Rehabil [Internet]. 2009 [cited 2020 Sep 3];23(6):522–33. Available from: https://pubmed.ncbi.nlm.nih.gov/19403556/

4. Curran GM, Bauer M, Mittman B, Pyne JM, Stetler C. Effectiveness-implementation hybrid designs: Combining elements of clinical effectiveness and implementation research to enhance public health impact. Med Care [Internet]. 2012 [cited 2019 Jul 24];50(3):217–26. Available from: https://www.ncbi.nlm.nih.gov/pmc/articles/PMC3731143/pdf/nihms480660.pdf

5. May CR, Mair F, Finch T, MacFarlane A, Dowrick C, Treweek S, et al. Development of a theory of implementation and integration: Normalization Process Theory. Implement Sci [Internet]. 2009 Dec 21 [cited 2020 Sep 3];4(1):29. Available from: http://implementationscience.biomedcentral.com/articles/10.1186/1748-5908-4-29

6. Powell BJ, Waltz TJ, Chinman MJ, Damschroder LJ, Smith JL, Matthieu MM, et al. A refined compilation of implementation strategies: Results from the Expert Recommendations for Implementing Change (ERIC) project. Implement Sci [Internet]. 2015;1 [cited 2020 Sep 24];10(1):21. Available from: http://implementationscience.biomedcentral.com/articles/10.1186/s13012-015-0209-1

### P45 ASSET-Ethiopia: Implementing the Ethiopian Primary Health Clinical Guidelines (EPHCG)

#### Alemayehu Bekele^1^, Atalay Alem^2^, Nadine Seward^3,4^, Tigist Eshetu^1^, Tewodros Haile^5^, Yeneneh Getachew^8^, Wondossen Mengistie^8^, Lara Fairall^4,6^, Nick Sevdalis^3,4^, Martin Prince^4^, Abebaw Fekadu^1,2^, Charlotte Hanlon^1,2,4,6^

##### ^1^Centre for Innovative Drug Development and Therapeutic Trials for Africa (CDT Africa), College of Health Sciences, Addis Ababa University, Addis Ababa, Ethiopia; ^2^Department of Psychiatry, WHO Collaborating Centre for Mental Health Research and Capacity Building, School of Medicine, College of Health Sciences, Addis Ababa University, Addis Ababa, Ethiopia; ^3^Centre for Implementation Science, Health Service and Population Research Department, Institute of Psychiatry, Psychology and Neuroscience, Kings College in London, UK; ^4^King’s Global Health Institute, King’s College London, UK; ^5^Department of Internal Medicine, School of Medicine, College of Health Sciences, Addis Ababa University, Addis Ababa, Ethiopia; ^6^Knowledge Translation Unit, University of Cape Town, Cape Town, South Africa; ^7^Centre for Global Mental Health, Health Service and Population Research Department, Institute of Psychiatry, Psychology and Neuroscience, Kings College in London, UK; ^8^Ministry of Health, Ethiopia

###### **Correspondence:** Alemayehu Bekele (alemayehubekele2002@gmail.com)

**Background**

The Ethiopian Primary Healthcare Clinical Guidelines (EPHCG) are a contextualized version of the evidence-aligned clinical algorithms for primary health care (PHC) clinicians in low- and middle-income countries, developed as part of the Practical Approach to Care Kit. In Ethiopia, implementation of EPHCG is being used to increase access to care for people with non-communicable disorders (NCDs) and mental health conditions (MHCs) [1,2]. The assessment aimed to explore barriers and enablers to the implementation of EPHCG.

**Method**

In-depth interviews were carried out with 10 PHC clinicians and one regional health administrator. EPHCG review meeting minutes were also used as a source of data. Contextual determinants were then mapped to the Consolidated Framework for Implementation Science (CFIR) and determinants of behaviours were mapped to the Theoretical Domains Framework (TDF). Potential implementation strategies were identified using the ERIC tool [3].

**Results**

The main contextual barriers associated with the implementation of EPHCG were the availability of resources (CFIR inner setting) including critical shortage of diagnostic tests and medication that undermined efforts to follow guideline-based care. There were significant barriers associated with the ability to address patient needs and resources (CFIR outer setting).

There were also several determinants of behaviours that influenced the ability to effectively implement EPHCG. For example, patients and the wider community had low awareness about NCDs/MHCs which resulted in patients presenting late with more severe illness. Table 1 describes the determinants for the implementation of EPHCG with potential implementation strategies.

**Conclusion**

The EPHCG implementation on expanding access to task-shared care for NCDs/MHCs has been constrained by various challenges. Health system bottlenecks need to be addressed to fully implement EPHCG to transform the quality of primary healthcare.

**References**

1. Ministry of Health. Ethiopian primary health care clinical guideline Implementation Manual. 2017

2. Ministry of Health. Ethiopian Primary healthcare clinical guidelines, 2017

3. Damschroder L, Aron D, Keith R, et al. Fostering implementation of health services research findings into practice: a consolidated framework for advancing implementation science. Implementation Science. 2009;4(1):50.

**Table 1 (abstract P45).** Barriers and enablers to the implementation of EPHCG identified using the Theoretical Domains Framework (TDF) and the Consolidated Framework for Implementation Research (CFIR)

Domain and determinant in relevant framework	Description	Implementation strategy identified using the ERIC tool
**CFIR framework**		
Intervention characteristics (evidence strength and quality, relative advantage)	PHC workers found the training offered for the EPHCG useful. The mode of workplace-based training for EPHCG was appreciated by some respondents as allowing more relevant and comprehensive discussion, but others preferred off-site training due to distractions and workplace pressures.	Strong policy directive supporting facility-based learning
Inner setting (readiness for implementation, available resources)	Critical shortage of diagnostic tests and medication which undermined efforts to follow the guideline, high patient load which made it difficult to consult the guidance and deliver comprehensive care, pressure on space and high turnover of staff.	Focus for Quality Improvement cycles
Implementation coverage	The EPHCG implementation was initiated in some primary healthcare facilities but not in others despite the conduct of cascade of training of trainers.	Strengthen supportive supervision and remove potential barriers
Outer setting (patient needs and resources)	The involvement of patients in making decisions about treatment options (person-centred care) was not commonly practiced by PHC workers.	Train PHC workers to develop competencies
**TDF**		
Knowledge	After training, knowledge PHC workers had improved knowledge post training that was reflected in ability to detect NCDs and MHCs. Post training, PHC workers still felt inadequately equipped to manage depression, diabetes mellitus and cardiac diseases. Patients and the wider community had low awareness about NCDs/MHCs which meant people presented late, with more severe illness, and were less engaged in self-management.	Supplementary training in mental health conditions – WHO’s mental health Gap Action Programme
Skills	Emotional support was not sufficiently provided to patients in need due to lack of skills among PHC workers	Train PHC workers to develop competencies
Confidence	PHC workers had improved confidence post training in managing hypertension and asthma. PHC workers felt inadequately equipped to manage depression, diabetes mellitus, and cardiac diseases	Information leaflets and community-based health extension worker detection of hypertension / awareness -raising
Intentions	Detection of NCDs and MHCs was not perceived to be a challenge. However, PHC clinicians reported that they were not proactive in managing care and monitoring adherence.	Train PHC workers to develop competencies
Follow up	Gaps in timely follow-up or tracking of patients were recognized and shortage of resources to use electronic devices for the tracking	Capacity development and mentoring support to PHC workers

### P46 Co-designing and co-conducting a multi-stakeholder implementation evaluation during the COVID-19 pandemic: a case study

#### Bridget Abell^1^, The CALD Covid Partnership Steering Group^2,3,4,5,6,7,8,9^

##### ^1^Australian Centre for Health Services Innovation (AusHSI) and Centre for Healthcare Transformation, School of Public Health & Social Work, Queensland University of Technology (QUT), Brisbane, Queensland, 4059, Australia; ^2^Metro South Hospital and Health Service, Brisbane, Queensland, 4122, Australia; ^3^Brisbane South Primary Health Network, Brisbane, Queensland, 4122, Australia; ^4^Multicultural Australia, Brisbane, Queensland, 4102, Australia; ^5^Access, Logan, Queensland, 4114, Australia; ^6^Queensland African Communities Council, Brisbane, Queensland, 4170, Australia; ^7^Refugee Health Network Queensland, Mater Hospital, Brisbane, Queensland, 4102, Australia; ^8^Australian Red Cross, Brisbane, Queensland, 4064, Australia; ^9^Queensland Program of Assistance to Survivors of Torture and Trauma, Brisbane, Queensland, 4102, Australia

###### **Correspondence:** Bridget Abell (bridget.abell@qut.edu.au)

**Background**

The COVID-19 pandemic has highlighted a need for collaborative, pragmatic, and low-resource methodologies for evaluation in implementation science. During the pandemic we designed and conducted a multi-stakeholder participatory evaluation of targeted efforts to engage with culturally and linguistically diverse (CALD) communities, which utilised such methods. This case study presents our learnings in the development and application of this approach.

**Method**

Our participatory approach involved collaboration between leaders, networks, and agencies representing CALD communities, health service stakeholders, and implementation scientists. Using virtual meetings and group emails, participants were stepped through the creation of an evaluation plan facilitated by logic model design. The logic model acted as a discussion tool to (a) frame evaluation goals, (b) articulate a theory of change, (c) identify questions about project implementation, and (d) determine how to ask them. Implementation scientists then facilitated mapping of this information to the RE-AIM framework [1] to identify evaluation measures and create data collection tools.

**Results**

The logic model was a feasible and simple template for co-designing implementation evaluation with implementation non-specialists. The process resulted in:

1. A shared understanding of program logic/theory of change
2. Key evaluation questions, mapped to the RE-AIM framework
3. A data collection matrix
4. A flexible interview guide/survey tool for adaptation by individual stakeholders into various formats and languages for data collection (e.g. online survey, in-person interview, phone call)
5. A plan for pragmatic and low-resource evaluation

Quantitative and qualitative data was successfully collected by members of the evaluation partnership with minimal training. This included interagency staff, bi-cultural workers, peer researchers, and CALD community leaders. Implementation academics performed data analysis.

**Conclusion**

We successfully co-designed and co-conducted a simple, flexible, framework-based implementation evaluation with non-specialists. The resulting lessons about stakeholder engagement and ownership of evaluation have applicability for implementation science beyond the pandemic.

**Reference**

**1.** Glasgow R, Vogt T, Boles S. Evaluating the public health impact of health promotion interventions: the RE-AIM framework. American journal of public health. 1999 Sep;89(9):1322-7.

### P47 What are the barriers and facilitators affecting the implementation of social robots for older adults and people with dementia? A scoping review

#### Wei Qi Koh^1^, Simone Anna Felding^2^, Kübra Beliz Budak^2^, Elaine Toomey^3^, Dympna Casey^1^

##### ^1^National University of Ireland Galway, H91 E3YV, Ireland; ^2^German Center for Neurodegenerative Diseases Witten, Stockumer Str. 12. 58452 Witten Germany; ^3^University of Limerick, V94 T9PX, Ireland

###### **Correspondence:** Wei Qi Koh (weiqi.koh@nuigalway.ie)

**Background**

Older adults and people with dementia are susceptible to psychosocial health issues, such as social isolation and loneliness among older adults and people with dementia. Social robots are a rapidly emerging field of technology that have been developed to address the psychosocial needs of this population. Little is known about the factors affecting their implementation in real-world practice. The aim of this review is to provide a systematic overview of the barriers and facilitators that has influenced the implementation of social robots for older adults and people with dementia.

**Method**

The Arksey and O’Malley framework with methodological enhancement by Levac et al, was used to guide the conduct of this review. Seven electronic databases were searched. Hand searching and backward citation tracing was conducted. Three independent reviewers were involved in screening and data charting. Findings were synthesised and categorised into the five domains outlined in the Consolidated Framework of Implementation Research (CFIR).

**Results**

53 studies were included in this review. Most social robots were used in participants’ homes and in care facilities. The determinants of implementation were mapped onto 18 constructs in the five domains of the CFIR. Barriers were most frequently mapped to the constructs in the domain “Intervention characteristics”, where issues such as complexity and technical obstacles impeded implementation. Most facilitators were mapped onto the domain “Patient needs and resources”. Studies have mostly focused on the internal validity (i.e. characteristics) of social robots, and there is significantly less studies which have investigated their external validity.

**Conclusion**

The breadth of evidence on the barriers and facilitators to the implementation of social robots for older adults and people with dementia was identified and synthesized. Moving forward, future research should place more focus on investigating contextual factors, using an implementation framework, to identify determinants and to guide the implementation of social robots.

**References**

Non applicable

### P48 The impact of Covid-19 on NCD-related scale up project implementation: Lessons from real-world case studies

#### Anusha R. Chander^1^, Rohina Joshi^2,3^, Amanda G. Thrift^1^

##### ^1^Department of Medicine, School of Clinical Sciences at Monash Health, Monash University, Melbourne, Australia; ^2^The George Institute for Global Health, University of New South Wales, Sydney, Australia; ^3^The George Institute, New Delhi, India

###### **Correspondence:** Anusha R. Chander (Anusha.Ramani-Chander@monash.edu)

**Background**

Little is known about the challenges in scaling up interventions targeting Non-Communicable diseases (NCDs), particularly in Low-and Middle-Income countries where the greatest burden lies.[1-4] Among 27 projects funded as part of the Global Alliance for Chronic Diseases call for scale-up studies of hypertension and diabetes,[5, 6] we aim to identify the impact of COVID-19 on the original project plans and the manner in which teams respond to this unanticipated implementation challenge.

**Methods**

We adopted a longitudinal, mixed-methods study design. First, we developed a data extraction tool, and interview guides, based on a literature review of scale up frameworks. We then reviewed project documents, such as protocols, and systematically captured details of the original scale up plans of each project using the extraction tool. Next, we undertook in-depth interviews with a range of stakeholders to gather diverse perspectives on the problems arising due to COVID-19 during early implementation, and any resulting adaptations made. The interviews are being open coded using thematic analysis.

**Results**

In preliminary findings from 21 projects (planning stage) and 39 interviews across 19 projects, we have identified that:

- There are many challenges faced by implementers including reorganised health priorities, redirection of government NCD resources, loss of communication with government health departments, covid-related restrictions, and greater consequences of COVID-19 in patients with NCD co-morbidities.
- The effects of COVID-19 on the interventions differ according to the nature and stage of the scale up, type of intervention, and nature of the collaboration.
- Some projects have stalled, while some investigators are engaging in supporting activities such as finalising training materials, and developing online workshops and training of staff.
- Few projects have identified new opportunities.

**Conclusion**

Studying the impact of COVID-19 on implementation of NCD-related scale up projects is providing important scientific insights that can assist planning of future scale up programs.

**Ethics**

This study was approved by the Monash University Human Research Ethics Committee (HREC #23482)

**Consent to publish**

Yes

**Acknowledgement**

We would like to acknowledge all the GACD Scale up research groups for their support and participation.

**References**

1. World Health Organization. Global Action Plan for the prevention and control of non-communicable diseases 2013-2020 [cited 2021 May 19]. Available from: https://www.who.int/publications/i/item/9789241506236.

2. NCD Countdown Collaborators. NCD Countdown 2030: pathways to achieving Sustainable Development Goal target 3.4. Lancet. 2020;396(10255):918-34.

3. Beaglehole R, Bonita R, Horton R, Adams C, Alleyne G, Asaria P, et al. Priority actions for the non-communicable disease crisis. Lancet. 2011;377(9775):1438-47.

4. World Health Organization. World Health Statistics: Monitoring health for the SDGs 2020 [cited 2021 May 19] Available from: https://www.who.int/publications/i/item/9789240005105.

5. Global Alliance for Chronic Diseases. List of projects that received research funding in the scale up call. 2019 [cited 2021 May 19] Available from: https://www.gacd.org/research-projects?diseases=scale-up&programme-countries=.

6. Global Alliance for Chronic Diseases. GACD 5th upscaling call - Hypertension and Diabetes. 2018 [cited 2021 May 19] Available from: https://www.gacd.org/funding/calls-for-proposals/gacd-scale-up-call.

### P49 Stakeholder relationships and governance mechanisms: Do these elements influence scale up project adaption during Covid-19?

#### Anusha R. Chander^1^, Rohina Joshi^2^, Amanda G. Thrift^1^

##### ^1^Department of Medicine, School of Clinical Sciences at Monash Health, Monash University, Melbourne, Australia; ^2^The George Institute for Global Health, University of New South Wales, Sydney, Australia; ^3^The George Institute, New Delhi, India

###### **Correspondence:** Anusha R. Chander (Anusha.Ramani-Chander@monash.edu)

**Background**

The increasing burden due to Non-Communicable diseases particularly in Low-and Middle-Income countries, needs to be addressed urgently by scaling up effective interventions.[1-5] Collaborative cross-country and multi-sectoral projects can assist in accelerating such efforts but there are challenges in managing such projects.[5, 6] We are applying systems thinking [7] approaches to study up to 27 funded scale up projects, targeting prevention, treatment and management of hypertension and/or diabetes.[8, 9] We will focus on the relationships that exists between multiple stakeholders in every project and the governance processes that help the project teams to manage and maintain these relationships. Our objective is to identify the manner in which these two elements influence each team’s ability to respond and adapt to challenges, such as Covid-19.

**Methods**

We first developed four separate semi-structured interview guides, based on a literature review of scale up frameworks. We interviewed a range of stakeholders from every project including high-income country (HIC) researchers, in-country researchers, implementors, industry partners, government representatives and staff. The interviews are being open coded using thematic analysis.

**Results**

In preliminary findings from 39 interviews across 19 scale up projects, we have identified that:

- Past relationships help establish trust between lead stakeholders.
- A strong, positive relationship between lead researchers located in HICs and in-country is a driving force for the collaboration.
- Building respectful relationships based on principles of equality between country research teams is essential for fostering a positive culture, encouraging open communication and increasing responsiveness.
- Multiple linkages between project teams and LMIC government stakeholders helps teams to problem solve and adapt to challenges.

**Conclusion**

Understanding the relationships and governance structures that exist in multi-stakeholder, multi-country collaborative projects and how these affect the ability of teams to adapt and be resilient to challenges such as Covid-19, can inform future planning of NCD-related scale up studies.

**Ethics**

This study was approved by the Monash University Human Research Ethics Committee (HREC #23482)

**Consent to publish**

Yes

**Acknowledgement**

We would like to acknowledge all the GACD Scale up research groups for their support and participation.

**References**

1. World Health Organization. Global Action Plan for the prevention and control of non-communicable diseases 2013-2020 [cited 2021 May 22]. Available from: https://www.who.int/publications/i/item/9789241506236.

2. NCD Countdown Collaborators. NCD Countdown 2030: pathways to achieving Sustainable Development Goal target 3.4. Lancet. 2020;396(10255):918-34.

3. Beaglehole R, Bonita R, Horton R, Adams C, Alleyne G, Asaria P, et al. Priority actions for the non-communicable disease crisis. Lancet. 2011;377(9775):1438-47.

4. Prabhakaran D, Singh K, Roth GA, Banerjee A, Pagidipati NJ, Huffman MD. Cardiovascular Diseases in India Compared With the United States. J Am Coll Cardiol. 2018;72(1):79-95.

5. Nishtar S, Niinisto S, Sirisena M, Vazquez T, Skvortsova V, Rubinstein A, et al. Time to deliver: report of the WHO Independent High-Level Commission on NCDs. Lancet. 2018;392(10143):245-52.

6. Olmen Jv, Delobelle P, Guwatudde D, Absetz P, Sanders D, Molsted Alvesson H, et al. Using a cross-contextual reciprocal learning approach in a multisite implementation research project to improve self-management for type 2 diabetes. BMJ Glob Health. 2018;3(6):e001068.

7. Savigny Dd, Taghreed A. Systems Thinking for Health Systems Strengthening: Alliance for Health Policy and Systems Research, WHO; 2009 [cited 2021 May 22]. Available from: https://www.who.int/alliance-hpsr/resources/9789241563895/en/.

8. Global Alliance for Chronic Diseases. List of projects that received research funding in the scale up call. 2019 [cited 2021 May 22] Available from: https://www.gacd.org/research-projects?diseases=scale-up&programme-countries=.

9. Global Alliance for Chronic Diseases. GACD 5th upscaling call - Hypertension and Diabetes. 2018 [cited 2021 May 22] Available from: https://www.gacd.org/funding/calls-for-proposals/gacd-scale-up-call.

